# Synthesis and structural characterization of four di­chlorido­bis­(cyclo­propyl­alkynyl­amidine)­metal complexes

**DOI:** 10.1107/S2056989018014895

**Published:** 2018-10-26

**Authors:** Sida Wang, Phil Liebing, Felix Engelhardt, Liane Hilfert, Sabine Busse, Frank T. Edelmann

**Affiliations:** a Chemisches Institut der Otto-von-Guericke-Universität Magdeburg, Universitätsplatz 2, 39106 Magdeburg, Germany

**Keywords:** amidinate ligand, amidine, manganese, iron, cobalt, crystal structure, hydrogen bonding

## Abstract

Deliberate hydrolysis of lithium cyclo­propyl­alkynylamidinates, Li[*c*-C_3_H_5_—C≡C(N*R*′)_2_] [*R*′ = ^*i*^Pr, Cy = cyclo­hex­yl)], afforded the hitherto unknown neutral cyclo­propyl­alkynyl­amidine derivatives *c*-C_3_H_5_—C≡C—C(N*R*′)(NH*R*′) [*R*′ = ^*i*^Pr (**1**), Cy (**2**)]. Subsequent reactions of **1** or **2** with metal(II) chlorides, *M*Cl_2_ (*M* = Mn, Fe, Co), provided the title complexes di­chlorido­bis­(3-cyclo­propyl-*N*,*N*′-diisopropyl­prop-2-ynamidine)­manganese(II), [MnCl_2_(C_12_H_20_N_2_)_2_], (**3**), di­chlorido­bis­(3-cyclo­propyl-*N*,*N*′-diisopropyl­prop-2-ynamidine)­iron(II), [FeCl_2_(C_12_H_20_N_2_)_2_], (**4**), di­chlorido­bis­(*N*,*N*′-di­cyclo­hexyl-3-cyclo­propyl­prop-2-ynamidine)­iron(II), [FeCl_2_(C_18_H_28_N_2_)_2_], (**5**), and di­chlorido­bis­(*N*,*N*′-di­cyclo­hexyl-3-cyclo­propyl­prop-2-ynamidine)­cobalt(II), [CoCl_2_(C_18_H_28_N_2_)_2_], (**6**), or more generally *M*Cl_2_[*c*-C_3_H_5_—C≡C—C(N*R*′)(NH*R*′)]_2_ [*R*′ = ^*i*^Pr, *M* = Mn (**3**), Fe (**4**); *R*′ = Cy, *M* = Fe (**5**), Co (**6**)] in moderate yields (30–39%). Besides their spectroscopic data (IR and MS) and elemental analyses, all complexes **3**–**6** were structurally characterized. The two isopropyl-substituted complexes **3** and **4** are isotypic, and so are the cyclo­hexyl-substituted complexes **5** and **6**. In all cases, the central metal atom is coordinated by two Cl atoms and two N atoms in a distorted-tetra­hedral fashion, and the structure is supported by intra­molecular N—H⋯Cl hydrogen bonds.

## Abstract 2

Transition-metal amidine complexes of the type *M*Cl_2_[*c*-C_3_H_5_—C≡C—C(N*R′*)(NH*R*′)]_2_ (*M* = Mn, Fe, Co) are readily available by a two-step synthesis starting from cyclo­proplacetyl­ene.

## Chemical context   

Over the past three decades, chelating anionic 1,3-di­aza­allyl-type ligands such as amidinates, [*R*C(N*R*′)_2_]^−^, and guanidin­ates, [*R*_2_NC(N*R*′)_2_]^−^, have gained tremendous importance in various fields of organometallic and coordination chemistry. These highly versatile *N*-chelating ligands are generally regarded as steric equivalents of the ubiquitous cyclo­penta­dienyl ligands (Collins, 2011 ▸; Edelmann, 2009 ▸, 2012 ▸, 2013 ▸). Unlike the closely related carboxyl­ate anions, [*R*CO_2_]^−^, the steric properties of amidinate anions can be tuned in a wide range by introducing different substituents at all three atoms of the NCN 1,3-di­aza­allyl unit. A rather inter­esting and potentially useful variation of the amidinate group is the use of alkinyl groups at the central C atom. Alkinyl­amidines of the composition *R*C≡C—C(=N*R*′)(N*R*′) are of inter­est because of their applications in organic synthesis (Ong *et al.*, 2006 ▸; Xu *et al.*, 2008 ▸; Weingärtner & Maas, 2012 ▸) and in biological and pharmacological systems (Rowley *et al.*, 2005 ▸; Sienkiewicz *et al.*, 2005 ▸). Moreover, alkinylamidinate complexes of transition metals and lanthanides effectively catalyze the addition of C—H, N—H and P—H bonds to carbodi­imides as well as the polymerization of polar monomers such as *∊*-caprolactone. Previously used alkynylamidinate anions have mainly included the *C*-phenyl and *C*-tri­methyl­silyl derivatives [*R*—C≡C—C(N*R*′)_2_]^−^ (*R* = Ph, SiMe_3_; *R*′ = ^*i*^Pr, Cy; Dröse *et al.*, 2010**a* ▸,b* ▸; Seidel *et al.*, 2012 ▸; Xu *et al.*, 2013 ▸).

We recently began with an investigation of alkinylamidinate ligands and complexes derived from cyclo­propyl­acetyl­ene. The cyclo­propyl group was selected because of its well-established electron-donating ability to an adjacent electron-deficient center. This way it is possible to electronically modify the amidinate ligand system rather than just changing its steric demand. In a first study, we described the synthesis and characterization of a series of lithium cyclo­propyl­ethinyl­amidinates, Li[*c*-C_3_H_5_—C≡C—C(N*R*′)_2_] [*R*′ = ^*i*^Pr, Cy (= cyclo­hex­yl)], which are readily accessible on a large scale using commercially available starting materials (cyclo­propyl­acetyl­ene, *N,N′*-diorganocarbodi­imides; Sroor *et al.*, 2013 ▸). Subsequently, these ligands have been employed for the preparation of new di- and trivalent lanthanide complexes (Sroor *et al.*, 2015*a* ▸,*b* ▸,*c* ▸,*d* ▸, 2016 ▸; Wang *et al.*, 2016 ▸). More recently, we became inter­ested in the chemistry of 3d metal complexes containing cyclo­propyl­ethinylamidinate ligands. In the course of this work, we occasionally observed and structurally characterized hydrolysis products of the composition *M*Cl_2_[*c*-C_3_H_5_—C≡C—C(N*R*′)(NH*R*′)] (*M* = Mn, Fe, Co; *R*′ = ^*i*^Pr, Cy), which contain the neutral amidines *c*-C_3_H_5_-C≡C—C(N*R*′)(NH*R*′) as new ligands. Neutral amidines are highly versatile ligands in coordination chemistry in their own right (Barker & Kilner, 1994 ▸; Coles, 2006 ▸). We report here the deliberate synthesis of two new cylo­propyl­alkynyl­amidines, *c*-C_3_H_5_—C≡C—C(N*R′*)(NH*R*′) (*R*′ = ^*i*^Pr, Cy) as well as the preparation and structural characterization of four first-row transition metal complexes of the type *M*Cl_2_[*c*-C_3_H_5_—C≡C—C(N*R*′)(NH*R*′)] (*M* = Mn, Fe, Co; *R*′ = ^*i*^Pr, Cy).

**Figure 1:**
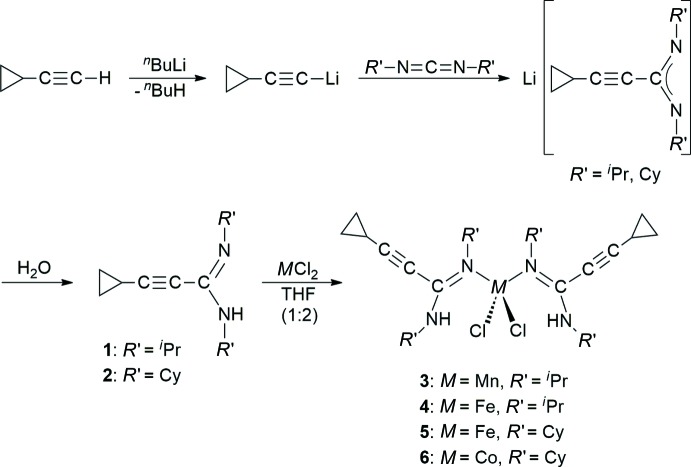
Bottom-up synthesis of the title compounds **3**–**6** starting from cyclo­propyl­acetyl­ene.

The title compounds were first discovered serendipitously when studying reactions of anhydrous metal(II) chlorides *M*Cl_2_ (*M* = Mn, Fe, Co) with 2 equiv. of the lithium cyclo­propyl­ethinylamidinates, Li[*c*-C_3_H_5_—C≡C—C(N*R*′)_2_] (*R*′ = ^*i*^Pr, Cy) in THF solution. Occasionally, small amounts of well-formed crystals were obtained, which turned out (by X-ray diffraction studies) to be the aforementioned hydrolysis products *M*Cl_2_[*c*-C_3_H_5_—C≡C—C(N*R*′)(NH*R*′)] (*M* = Mn, Fe, Co; *R*′ = ^*i*^Pr, Cy). We then decided to prepare these complexes in a deliberate manner. As illustrated in Fig. 1 ▸, the bottom-up synthesis starts with the readily available lithium cyclo­propyl­ethinylamidinates, Li[*c*-C_3_H_5_—C≡C—C(N*R*′)_2_] (*R*′ = ^*i*^Pr, Cy; Sroor *et al.*, 2013 ▸), which were made by addition of *c*-C_3_H_5_—C≡C—Li (prepared *in situ* from cyclo­propyl­acetyl­ene and ^*n*^BuLi) to the carbodiimides *R*′—N=C=N—*R*′ (*R* = ^*i*^Pr, Cy). The lithium amidinate inter­mediates were then carefully hydrolyzed under controlled conditions to afford the neutral amidines *c*-C_3_H_5_—C≡C—C(N*R*′)(NH*R*′) [*R*′ = ^*i*^Pr (**1**), Cy (**2**)] in >70% isolated yields. Both compounds form yellow oils, which were characterized by the usual set of spectroscopic data (MS, ^1^H NMR, ^13^C NMR, IR) and elemental analysis. With the free amidine ligands in hand, the metal complexes with first-row transition metals could easily be prepared by treatment of metal(II) chlorides *M*Cl_2_ (*M* = Mn, Fe, Co) with 2 equiv. of either **1** or **2** in THF solution. The manganese(II) complex **3** as well as the two iron(II) complexes **4** and **5** form colourless crystals, while the cobalt(II) complex **6** is blue. The compositions of all four products as 1:2 complexes were confirmed by elemental analyses. The title compounds **3**–**6** were also characterized by their IR and mass spectra. The mass spectra showed a number of readily inter­pretable peaks resulting *e.g.* from loss of one amidine ligand or one or both chlorine atoms. IR bands in the region above *ca* 3100 cm^−1^ could be assigned to the ν(N—H) vibrations, while strong bands around 1570 cm^−1^ were characteristic for the C=N double bond in the amidine ligands. In the far-infrared region, the *M*—Cl bands could be clearly assigned by comparison with literature values (Clark & Williams, 1965 ▸; Takemoto *et al.*, 1974 ▸) and IR spectra of the respective anhydrous metal(II) chlorides, *M*Cl_2_ (*M* = Mn, Fe, Co; for details see the *Synthesis and crystallization* section).
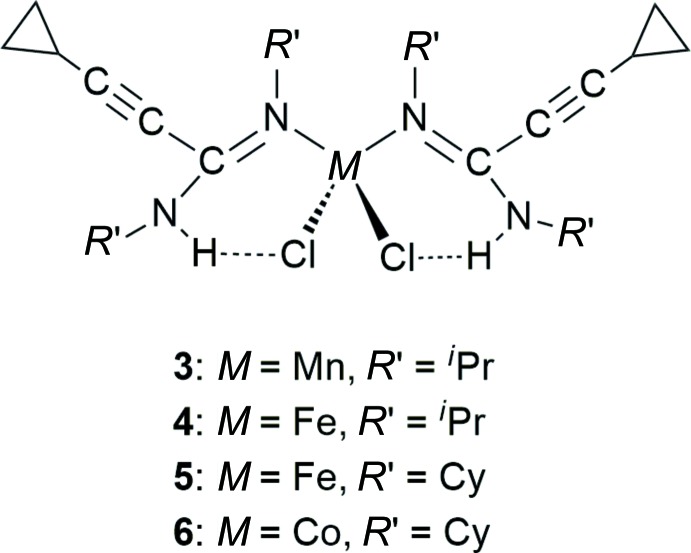


## Structural commentary   

**Figure 2:**
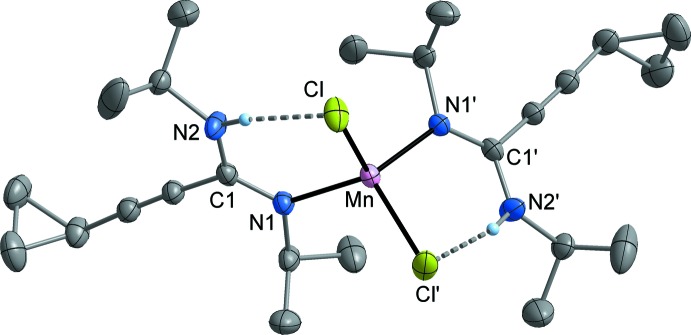
Mol­ecular structure of **3** in the crystal. Displacement ellipsoids are drawn at the 50% level, C-bound H atoms omitted for clarity. Symmetry code: (′) 1 − *x*, 1 − *y*, *z*.

**Figure 3:**
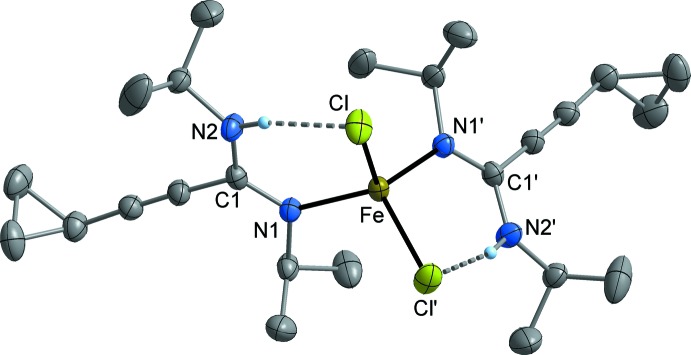
Mol­ecular structure of **4** in the crystal. Displacement ellipsoids are drawn at the 50% level, C-bound H atoms omitted for clarity. Symmetry code: (′) 1 − *x*, 1 − *y*, *z*.

**Figure 4:**
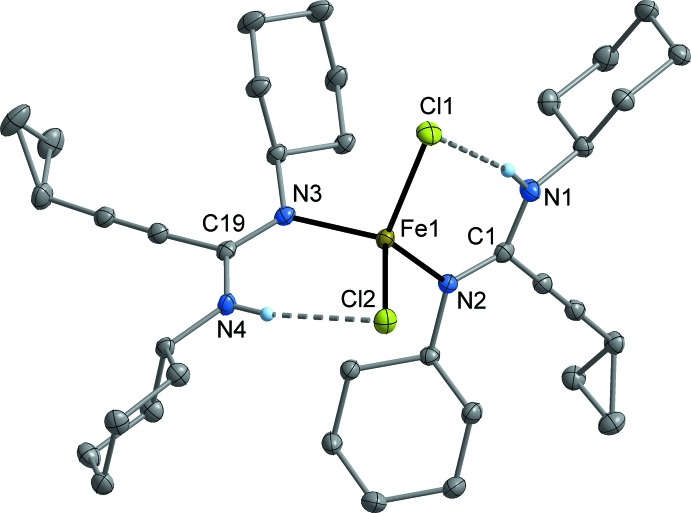
Mol­ecular structure of **5** in the crystal. Displacement ellipsoids are drawn at the 50% level, C-bound H atoms omitted for clarity.

**Figure 5:**
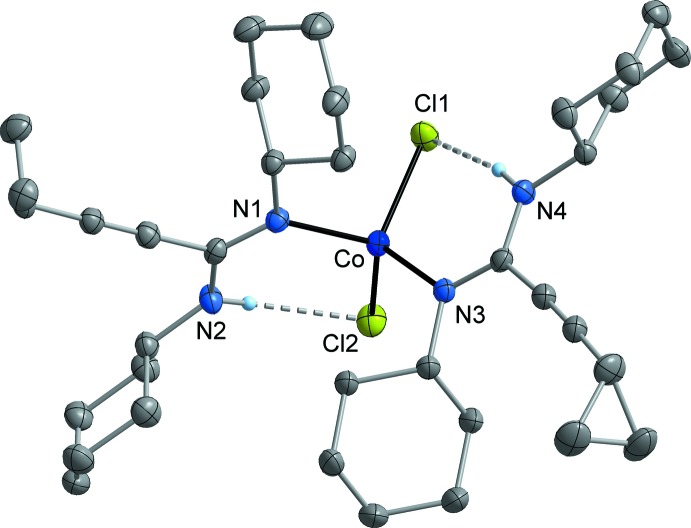
Mol­ecular structure of **6** in the crystal. Displacement ellipsoids are drawn at the 50% level, C-bound H atoms omitted for clarity.

MnCl_2_[*c*-C_3_H_5_—C≡C—C(N^*i*^Pr)(NH^*i*^Pr)]_2_ (**3**; Fig. 2 ▸) and FeCl_2_ [*c*-C_3_H_5_—C≡C—C(N^*i*^Pr)(NH^*i*^Pr)]_2_ (**4**; Fig. 3 ▸) crystallize isotypic­ally in the ortho­rhom­bic space group *Fdd*2. The metal atom is situated on a crystallographic twofold axis and is surrounded by two symmetry-equivalent chlorido ligands and two symmetry-equivalent amidine ligands. The latter are attached to the metal atom in a monodentate κ*N* mode *via* the non-protonated nitro­gen atom (N1). The N—H moiety is involved in an intra­molecular N—H⋯Cl bond (Tables 1 ▸ and 2 ▸). The crystal structures of FeCl_2_[*c*-C_3_H_5_—C≡C—C(NCy)(NHCy)]_2_ (**5**; Fig. 4 ▸) and CoCl_2_[*c*-C_3_H_5_-C≡C—C(NCy)(NHCy)]_2_ (**6**; Fig. 5 ▸) are isotypic in the monoclinic space group *P*2_1_/*c*. In this case, the two amidine ligands are not symmetry-equivalent, but nonetheless the mol­ecular structures resemble those of **3** and **4**.

Compound **3** represents a rare example of a complex of tetra-coordinated manganese with nitro­gen ligands, while a larger number of the corresponding iron and cobalt complexes are known. The Mn—N bond length in **3** is 2.160 (2) Å and therefore comparable with literature data (Handley *et al.*, 2001 ▸; Wang, 2009 ▸). In the iron complexes, the Fe—N distances are very similar at 2.088 (3) Å (**4**), and 2.073 (2)–2.079 (2) Å (**5**). These values are in the range of Fe—N distances usually observed in *M*Cl_2_ *L*_2_-type complexes, where *L* is a ligand with an *sp* ^2^-hybridized nitro­gen donor (Benson *et al.*, 2010 ▸; Xiao *et al.*, 2011 ▸; Batcup *et al.*, 2014 ▸). The same is true for the cobalt complex **6**, having Co—N bond lengths of 2.041 (2) and 2.043 (2) Å (Riggio *et al.*; 2001 ▸; Jian *et al.*, 2003 ▸; Xiao *et al.*, 2011 ▸). The set of C—N bond lengths within the NCN group of the amidine ligands is virtually equal in **3**–**6**, including one formal C=N double bond at 1.309 (2)–1.315 (4) Å, and one formal C—N single bond at 1.337 (4)–1.340 (2) Å. The small difference between single- and double-bond length may indicate some degree of delocalization of the π-electron density. The observed values are consistent with other metal complexes having metal-coordinated amidine moieties (Dröse *et al.*, 2010**a* ▸,b* ▸; Harmgarth *et al.*, 2014 ▸, 2017**a* ▸,b* ▸; Hillebrand *et al.*, 2014 ▸). The hydrogen-bonded N⋯Cl separations are similar in **3**–**6**, being in the narrow range of 3.175 (3)–3.251 (2) Å (Tables 1 ▸–4 ▸ ▸ ▸).

## Supra­molecular features   

All four title compounds **3**–**6** display weak intra- and inter­molecular C—H⋯Cl contacts (Tables 1 ▸–4 ▸ ▸ ▸) involving the *cyclo*-propyl and *iso*-propyl or *cyclo*-hexyl groups, respectively.

## Chemistry of related structures   

For reviews on the coordination chemistry of neutral amidines, see Barker & Kilner (1994 ▸) and Coles (2006 ▸).

## Synthesis and crystallization   

**General Procedures:** All reactions were carried out in oven-dried or flame-dried glassware in an inert atmosphere of dry argon employing standard Schlenk and glovebox techniques. The solvent THF was distilled from sodium/benzo­phenone in a nitro­gen atmosphere prior to use. *n*-Butyl­lithium (1.6 *M* in hexa­nes) was purchased from Sigma–Aldrich. ^1^H NMR (400 MHz) and ^13^C NMR (100.6 MHz) spectra were recorded in THF-*d_8_* solutions using a Bruker DPX 400 spectrometer at 298 K. Chemical shifts are referenced to tetra­methyl­silane. IR spectra were measured with a Bruker Vertex 70V spectrom­eter equipped with a diamond ATR unit between 4000 and 50 cm^−1^. The relative intensities of the absorption bands are given as very strong (*vs*), strong (*s*), medium (*m*), weak (*w*) and shoulder (*sh*). Electron impact mass spectra were measured on a MAT95 spectrometer with an ionization energy of 70 eV. Microanalyses of the compounds were performed using a vario EL cube apparatus from Elementar Analysensysteme GmbH.

**Synthesis of 3-cyclo­propyl-** ***N***,***N***′**-diiso­propyl­propynamidine**, ***c*** **-C_3_H_5_—C**≡**C—C(N** ***^i^*** **Pr)(NH** ***^i^*** **Pr) (1):** A THF (80 ml) solution of cyclo­propyl­acetyl­ene (4.2 ml, 50 mmol) in a Schlenk flask (250 ml) was cooled to 253 K and treated slowly with *n*-butyl­lithium (50 mmol, 1.6 *M* solution in hexa­nes). After 30 min, neat *N*,*N′*-diiso­propyl­carbodi­imide (7.8 ml, 50 mmol) was added and the mixture was stirred for 15 min at 253 K. The solution was warmed to room temperature and stirred for 1 h. During this time, the solution colour turned yellow. 20 ml of distilled water were added and stirring was continued for 30 min. The solution was separated using a separatory funnel and allowed to stand overnight after adding 3.0 g of anhydrous magnesium sulfate to remove the remaining water. The solvents were removed under vacuum to obtain **1** as a yellow oil. Yield: 6.9 g, 72%. Elemental analysis for C_12_H_20_N_2_ (192.30 g mol^−1^): C, 74.95; H, 10.48; N, 14.57; found C, 74.74; H, 10.46; N, 14.58. MS (EI, *M* = 192.30): *m*/*z* (%) 107.04 (10) [*M* – 2^*i*^Pr]^+^, 149.11 (68) [*M* − ^*i*^Pr]^+^, 164.12 (47) [*M* − 2CH_3_]^+^, 177.13 (100) [*M* − CH_3_]^+^, 191.14 (43) [*M*]^+. 1^H NMR (400.1 MHz, THF-*d_8_*, 298 K): *δ* (ppm) 4.71–4.78 (*br*, 1H, N*H*, NHCN), 3.72–3.88 (*s*, 2H, C*H*, ^*i*^Pr), 1.31–1.38 (*m*, 1H, C*H*, *c*-C_3_H_5_), 0.97–1.04 (*d*, 12H, C*H*_3_, ^*i*^Pr), 0.79–0.84 (*m*, 4H, C*H*_2_, *c*-C_3_H_5_), 0.66–0.69 (*m*, 4H, C*H*_2_, *c*-C_3_H_5_). ^13^C NMR (100.6 MHz, THF-*d_8_*, 298 K): *δ* (ppm) 140.5 (NH*C*N), 96.6 (CH—*C*≡C), 69.2 (C≡*C*—C), 67.8 (*C*H, ^*i*^Pr), 26.8 (*C*H_3_, ^*i*^Pr), 9.83 (*C*H_2_, *c*-C_3_H_5_), 0.37 (*C*H, *c*-C_3_H_5_). IR (ATR): *ν* (cm^−1^) 3440 (*w*, N—H), 3415 (*w*, N—H), 3096 (*w*), 3014 (*w*), 2963 (*s*, C—H), 2931 (*m*), 2867 (*m*, C—H), 2614 (*w*), 2226 (*m*), 1606 (*vs*, N=C), 1487 (*m*), 1466 (*m*), 1453 (*m*), 1375 (*m*), 1360 (*m*), 1344 (*m*), 1317 (*m*), 1263 (*m*), 1178 (*m*), 1132 (*m*), 1088 (*w*), 1055 (*w*), 1031 (*w*), 970 (*w*), 943 (*m*), 880 (*w*), 849 (*w*), 812 (*w*), 685 (*m*), 616 (*w*), 472 (*w*), 424 (*w*), 254 (*w*), 105 (*w*), 71 (*w*), 60 (*w*).

**Synthesis of 3-cyclo­propyl-** ***N***,***N***′**-di­cyclo­hexyl­propyn­amid­ine**, ***c*** **-C_3_H_5_—C**≡**C—C(NCy)(NHCy) (2):** A THF (100 ml) solution of cyclo­propyl­acetyl­ene (4.2 ml, 50 mmol) in a Schlenk flask (250 ml) was cooled to 253 K and treated slowly with *n*-butyl­lithium (50 mmol, 1.6 *M* solution in hexa­nes). After 30 min, *N*,*N′*-di­cyclo­hexyl­carbodi­imide (10.3 g, 50 mmol) was added and the rest of the reaction mixture was worked up as described for **1**. The solvent was removed under vacuum to obtain **2** as a yellow oil. Yield: 10.1 g, 74%. Elemental analysis for C_18_H_28_N_2_ (272.43 g mol^−1^): C, 79.36; H, 10.36; N, 10.28; found C, 79.36; H, 10.30; N, 10.38. MS (EI, *M* = 272.40): *m*/*z* (%) 109.06 (19) [*M* − 2Cy]^+^, 189.13 (75) [*M* − Cy]^+^, 272.23 (79) [*M*]^+. 1^H NMR (400.1 MHz, THF-*d_8_*, 293 K): δ (ppm) 4.87–4.95 (*s*, 1H, N*H*CN), 1.69–1.06 (*m*, 20H, C*H*_2_, Cy), 1.40–1.34 (*m*, 1H, C*H*, *c*-C_3_H_5_), 0.79–0.86 (*m*, 2H, C*H*_2_, *c*-C_3_H_5_), 0.61–0.69 (*m*, 2H, C*H*_2_, *c*-C_3_H_5_). ^13^C NMR (100.6 MHz, THF-*d_8_*, 298 K): δ (ppm) 141.5 (NH*C*N), 95.6 (CH—*C*≡C), 69.2 (C≡*C*—C), 64.5 (*C*H, Cy), 25.1–26.8 (*C*H_2_, Cy), 8.83 (*C*H_2_, *c*-C_3_H_5_), 0.37 (*C*H, *c*-C_3_H_5_). IR (ATR): *ν* (cm^−1^) 3351 (*w*, N—H), 3062 (*w*), 2960 (*vs*, C—H), 2925 (*s*), 2866 (*m*, C—H), 2225 (*w*), 2116 (*w*), 1917 (*w*), 1855 (*w*), 1796 (*w*), 1661 (*w*), 1626 (*m*, N=C), 1601 (*m*), 1591 (*m*), 1530 (*w*), 1382 (*m*), 1361 (*m*), 1330 (*m*), 1314 (*m*), 1255 (*s*), 1177 (*m*), 1162 (*m*), 1146 (*m*), 1107 (*m*), 1058 (*m*), 1043 (*m*), 972 (*w*), 956 (*w*), 923 (*m*), 888 (*w*), 865 (*w*), 839 (*w*), 819 (*m*), 794 (*s*), 753 (*vs*), 706 (*w*), 678 (*m*), 622 (*w*), 601 (*w*), 577 (*w*), 527 (*w*), 519 (*w*), 465 (*w*), 441 (*m*), 416 (*m*), 326 (*s*), 275 (*s*), 169 (*m*), 152 (*m*), 114 (*m*), 88 (*m*), 57 (*w*).

**Synthesis of di­chlorido­bis­(3-cyclo­propyl-** ***N***,***N*** **′-diiso­propyl­prop-2-ynamidine)­manganese(II), MnCl_2_[** ***c*** **-C_3_H_5_—C**≡**C—C(N** ***^i^*** **Pr)(NH** ***^i^*** **Pr)]_2_ (3):** A solution of anhydrous MnCl_2_ (0.33 g, 2.6 mmol) in 30 ml of THF was added to a solution of **1** (1.0 g, 5.2 mmol) in 50 ml of THF. The reaction mixture was heated to 333 K by water bath and stirred at room temperature for 12 h, resulting in a brown suspension. The filtrate was concentrated to *ca* 10 ml. Crystallization at r.t. afforded **3** as colourless crystals. Yield: 0.52 g, 39%. M.p. = 395 K. Elemental analysis for C_24_H_40_Cl_2_MnN_4_ (510.45 g mol^−1^): C, 56.47; H, 7.90; N, 10.98; found C, 56.49; H, 7.93; N, 10.98. MS (EI, *M* = 510.45): *m*/*z* (%) 425.2 (50) [*M* − 2Cl − CH_3_]^+^, 433.2 (2) [*M* − Cl – ^*i*^Pr]^+^, 498.2 (100) [*M* − CH_2_ + 2H]^+^. IR (ATR): *ν* (cm^−1^) 3411 (*w*, N—H), 3239 (*m*, N—H), 3129 (*w*, N—H), 2967 (*m*), 2930 (*w*), 2872 (*w*), 2217 (*s*), 1628 (*w*), 1571 (*vs*, N=C), 1464 (*s*), 1432 (*vs*), 1382 (*w*), 1363 (*m*), 1330 (*m*), 1313 (*m*), 1243 (*m*), 1172 (*m*), 1132 (*vs*), 1061 (*w*), 1032 (*w*), 963 (*s*), 940 (*w*), 879 (*w*), 843 (*m*), 831 (*m*), 705 (*s*), 658 (*m*), 603 (*w*), 520 (*w*), 489 (w), 460 (*w*), 387 (w), 333 (*m*), 279 (*vs*, Mn—Cl), 207 (*m*), 173 (*m*), 128 (*vs*).

**Synthesis of di­chlorido­bis­(3-cyclo­propyl-** ***N***,***N*** **′-diiso­propyl­prop-2-ynamidine)­iron(II), FeCl_2_[** ***c*** **-C_3_H_5_—C**≡**C—C(N** ***^i^*** **Pr)(NH** ***^i^*** **Pr)]_2_ (4):** A solution of anhydrous FeCl_2_ (0.33 g, 2.6 mmol) in 30 ml of THF was added to a solution of **1** (1.0 g, 5.2 mmol) in 50 ml of THF following the procedure given for **3**. Crystallization at room temperature afforded **4** as colourless crystals. Yield: 0.40 g, 30%. M.p. = 400 K. Elemental analysis for C_24_H_40_Cl_2_FeN_4_ (511.35 g mol^−1^): C, 56.37; H, 7.88; N, 10.96; found C, 56.34; H, 7.75; N, 10.98%. MS (EI, *M* = 511.35): *m*/*z* (%) 432.4 (100) [*M* − Cl − ^*i*^Pr]^+^, 439.1 (40) [*M* − 2Cl]^+^, 475.4 (63) [*M* − Cl]^+^, 501.0 (100) [*M* − CH_2_ + 2H]^+^. IR (ATR): *ν* (cm^−1^) 3290 (*w*, N—H), 3222 (*w*, N—H), 3119 (*w*, N—H), 2976 (*m*, C—H), 2933 (*w*), 2874 (*w*, C—H), 2225 (*m*), 1619 (*s*), 1568 (*m*, N=C), 1485 (*w*), 1463 (*w*), 1429 (*w*), 1392 (*w*), 1372 (*w*), 1309 (*w*), 1244 (*w*), 1169 (*m*), 1129 (*m*), 1062 (*w*), 1033 (*w*), 963 (*m*), 939 (*m*), 879 (*m*), 846 (*m*), 818 (*w*), 793 (*w*), 709 (*s*), 691 (*s*), 649 (*s*), 599 (s), 520 (*s*), 460 (*s*), 353 (*vs*), 313 (*vs*), 280 (*vs*), 211 (*vs*, Fe—Cl), 134 (*s*), 68 (*s*).

**Synthesis of di­chlorido­bis­(** ***N***,***N*** **′-di­cyclo­hexyl-3-cyclo­prop­yl­prop-2-ynamidine)­iron(II), FeCl_2_[** ***c*** **-C_3_H_5_-C**≡**C—C(NCy)(NHCy)]_2_ (5):** A solution of anhydrous FeCl_2_ (0.23 g, 1.8 mmol) in 30 ml of THF was added to a solution of **2** (1.0 g, 3.6 mmol) in 50 ml of THF. The reaction mixture was heated to 333 K by water bath and stirred at room temperature for 12 h, resulting in a brown suspension. The filtrate was concentrated to *ca* 10 ml. Crystallization at 278 K afforded **5** in the form of colorless crystals. Yield: 0.45 g, 37%. M.p. = 405 K. Elemental analysis for C_36_H_56_Cl_2_FeN_4_ (671.61 g mol^−1^): C, 65.66; H, 8.21; N, 8.57; found C, 64.38; H, 8.40; N, 8.34%. MS (EI, *M* = 671.61): *m*/*z* (%) 363.17 (24) [*M* − *c*-C_3_H_5_—C≡C—C(NCy)(NHCy) − Cl]^+^, 457.08 (74) [*M* − 3C_3_H_7_ − C_6_H_11_]^+^, 540.13 (100) [*M* − 3C_3_H_7_]^+^. IR (ATR): *ν* (cm^−1^) 3214 (*w*, N—H), 2928 (*s*, C—H), 2852 (*s*, C—H), 2227 (*s*), 1573 (*vs*, N=C), 1448 (*s*), 1365 (*m*), 1347 (*w*), 1308 (*w*), 1245 (*m*), 1188 (*w*), 1154 (*w*), 1062 (*w*), 1031 (*w*), 974 (*m*), 891 (*w*), 858 (*w*), 842 (*w*), 814 (*w*), 702 (*m*), 603 (*w*), 549 (*w*), 474 (*w*), 443 (*w*), 279 (*s*), 198 (*vs*, Fe—Cl), 140 (*s*), 121 (*s*), 107 (*s*), 89 (*m*).

**Synthesis of di­chlorido­bis­(** ***N***,***N*** **’-di­cyclo­hexyl-3-cyclo­propyl­prop-2-ynamidine)­cobalt(II), CoCl_2_[** ***c*** **-C_3_H_5_-C**≡**C—C(NCy)(NHCy)]_2_CoCl_2_ (6):** A solution of anhydrous CoCl_2_ (0.23 g, 1.8 mmol) in 30 ml of THF was added to a solution of **2** (1.0 g, 3.6 mmol) in 50 ml of THF following the procedure given for **5**. Crystallization at 278 K afforded **6** in the form of blue crystals. Yield: 0.45 g, 37%. M.p. = 399 K. Elemental analysis for C_36_H_56_Cl_2_CoN_4_ (674.69 g mol^−1^): C, 64.09; H, 8.37; N, 8.30; found C, 63.69; H, 8.31; N, 9.26%. MS (EI, *M* = 674.69): *m*/*z* (%) 402.23 (24) [*M* − *c*-C_3_H_5_—C≡C—C(NCy)(NHCy)]^+^, 461.32 (89) [*M* − 3C_3_H_7_ − C_6_H_11_]^+^, 544.39 (15) [*M* − 3C_3_H_7_]^+^. IR (ATR): *ν* (cm^−1^) 3440 (*w*, N—H), 3212 (*w*, N—H), 3128 (*w*, N—H), 3090 (*w*), 3008 (*w*), 2925 (*vs*, C—H), 2850 (*s*, C—H), 2662 (*w*), 2228 (*m*), 1690 (*w*), 1635 (*w*), 1605 (*m*), 1575 (N=C), 1486 (*m*), 1447 (*vs*), 1433 (*s*), 1363 (*s*), 1346 (*m*), 1300 (*w*), 1257 (*m*), 1221 (*w*), 1188 (*w*), 1157 (*w*), 1090 (*w*), 1064 (*m*), 1031 (*m*), 973 (*m*), 889 (*w*), 858 (*m*), 841 (*w*), 815 (*w*), 788 (*w*), 701 (*s*), 656 (*m*), 549 (*w*), 475 (*w*), 444 (*w*), 430 (*w*), 392 (*w*), 349 (*w*), 292 (*vs*, Co—Cl), 228 (*m*), 204 (*w*), 166 (*w*), 127 (*vs*), 74 (*w*).

For comparison, the far infrared spectra of the anhydrous metal dichlorides *M*Cl_2_ (*M* = Mn, Fe, Co) were also measured:

IR (KBr): *ν* MnCl_2_ (cm^−1^) 1064 (*w*), 1230 (*w*), 492 (*w*), 434 (*w*), 318 (*w*), 163 (*vs*, Mn—Cl) , 83 (*s*), 64 (*s*).

IR (KBr): *ν* FeCl_2_ (cm^−1^) 3461 (*w*), 2977 (*w*), 2113 (*w*), 1993 (*w*), 1599 (*w*), 1389 (*w*), 1096 (*w*), 931 (*w*), 812 (*w*), 330 (*w*), 144 (*vs*, Fe—Cl), 54 (*s*).

IR (KBr): *ν* CoCl_2_ (cm^−1^) 1599 (*w*), 615 (*w*), 348 (*w*), 189 (*vs*, Co—Cl).

X-ray quality single crystals of complexes **3**–**6** were obtained at r.t. from concentrated solutions in THF.

## Refinement   

Crystal data, data collection and structure refinement details are summarized in Table 5 ▸. H atoms attached to C atoms were fixed geometrically and refined using a riding model. The CH_3_ groups in **3** and **4** were allowed to rotate freely around the C—C vector, the corresponding C—H distances were constrained to 0.98 Å. C—H distances within CH_2_ groups were constrained to 0.99 Å, C—H distances within CH groups to 1.00 Å. H atoms attached to N atoms were located in the difference-Fourier map and refined, the N—H distances were restrained to 0.88 (2) Å. The *U*_iso_(H) values were set at 1.5*U*_eq_(C) for the methyl groups in **3** and **4**, and at 1.2*U*_eq_(*X*) (*X* = C, N) in all other cases. For **6**, the reflections (011) and (002) disagreed strongly with the structural model and were therefore omitted from the refinement.

## Supplementary Material

Crystal structure: contains datablock(s) 3, 4, 5, 6. DOI: 10.1107/S2056989018014895/zl2740sup1.cif

Structure factors: contains datablock(s) 3. DOI: 10.1107/S2056989018014895/zl27403sup2.hkl

Structure factors: contains datablock(s) 4. DOI: 10.1107/S2056989018014895/zl27404sup3.hkl

Structure factors: contains datablock(s) 5. DOI: 10.1107/S2056989018014895/zl27405sup4.hkl

Structure factors: contains datablock(s) 6. DOI: 10.1107/S2056989018014895/zl27406sup5.hkl

CCDC references: 1848876, 1848879, 1848878, 1848877

Additional supporting information:  crystallographic information; 3D view; checkCIF report

## Figures and Tables

**Table 1:** Hydrogen-bond geometry (Å, °) for **3** [Chem scheme1]

*D*—H⋯*A*	*D*—H	H⋯*A*	*D*⋯*A*	*D*—H⋯*A*
N2—H1⋯Cl	0.85 (2)	2.36 (2)	3.197 (3)	170 (3)
C8—H9⋯Cl^i^	0.98	2.88	3.776 (4)	152
C5—H4⋯Cl^ii^	0.99	2.95	3.931 (4)	172
C10—H14⋯Cl^ii^	1.00	2.93	3.643 (3)	129
C4—H2⋯Cl^iii^	1.00	2.67	3.516 (3)	143

Symmetry codes: (i) 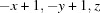; (ii) 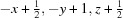; (iii) 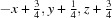.

**Table 2:** Hydrogen-bond geometry (Å, °) for **4** [Chem scheme1]

*D*—H⋯*A*	*D*—H	H⋯*A*	*D*⋯*A*	*D*—H⋯*A*
N2—H1⋯Cl	0.87 (2)	2.32 (3)	3.175 (3)	169 (4)
C8—H9⋯Cl^i^	0.98	2.84	3.728 (5)	151
C5—H4⋯Cl^ii^	0.99	2.98	3.963 (5)	172
C10—H14⋯Cl^ii^	1.00	2.98	3.679 (4)	128
C4—H2⋯Cl^iii^	1.00	2.68	3.510 (4)	140

Symmetry codes: (i) 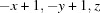; (ii) 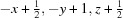; (iii) 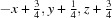.

**Table 3:** Hydrogen-bond geometry (Å, °) for **5** [Chem scheme1]

*D*—H⋯*A*	*D*—H	H⋯*A*	*D*⋯*A*	*D*—H⋯*A*
N2—H1⋯Cl2	0.84 (2)	2.42 (2)	3.2511 (19)	169 (2)
N4—H29⋯Cl1	0.85 (2)	2.41 (2)	3.2459 (18)	170 (2)
C22—H30⋯Cl1^i^	1.00	2.90	3.744 (3)	143
C35—H53⋯Cl1^i^	0.99	3.05	3.613 (2)	118
C28—H40⋯Cl2^ii^	0.99	2.91	3.699 (2)	138

Symmetry codes: (i) 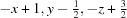; (ii) 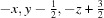.

**Table 4:** Hydrogen-bond geometry (Å, °) for **6** [Chem scheme1]

*D*—H⋯*A*	*D*—H	H⋯*A*	*D*⋯*A*	*D*—H⋯*A*
N2—H1⋯Cl2	0.85 (2)	2.37 (2)	3.1979 (16)	166 (2)
N4—H29⋯Cl1	0.86 (2)	2.35 (2)	3.1917 (15)	168 (2)
C22—H30⋯Cl1^i^	1.00	2.95	3.800 (2)	144
C33—H49⋯Cl1^i^	0.99	3.09	3.628 (2)	115
C28—H40⋯Cl2^ii^	0.99	2.96	3.758 (2)	139

Symmetry codes: (i) 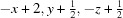; (ii) 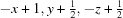.

**Table 5:** Experimental details

	**3**	**4**	**5**	**6**
Crystal data				
Chemical formula	[MnCl_2_(C_12_H_20_N_2_)_2_]	[FeCl_2_(C_12_H_20_N_2_)_2_]	[FeCl_2_(C_18_H_28_N_2_)_2_]	[CoCl_2_(C_18_H_28_N_2_)_2_]
*M*_r_	510.44	511.35	671.59	674.67
Crystal system, space group	Orthorhombic, *F* *d* *d*2	Orthorhombic, *F* *d* *d*2	Monoclinic, *P*2_1_/*c*	Monoclinic, *P*2_1_/*c*
Temperature (K)	153	153	100	153
*a*, *b*, *c* (Å)	17.6701 (10), 30.9809 (19), 10.1452 (5)	17.5703 (9), 30.9167 (12), 10.1110 (6)	13.905 (7), 12.500 (6), 20.742 (11)	13.8898 (3), 12.5574 (3), 20.8394 (5)
α, β, γ (°)	90, 90, 90	90, 90, 90	90, 92.24 (4), 90	90, 91.717 (2), 90
*V* (Å^3^)	5553.8 (5)	5492.5 (5)	3603 (3)	3633.17 (15)
*Z*	8	8	4	4
Radiation type	Mo *K*α	Mo *K*α	Mo *K*α	Mo *K*α
μ (mm^−1^)	0.69	0.76	0.60	0.65
Crystal size (mm)	0.33 × 0.24 × 0.10	0.27 × 0.25 × 0.25	0.26 × 0.19 × 0.12	0.39 × 0.19 × 0.10

Data collection				
Diffractometer	Stoe IPDS 2T	Stoe IPDS 2T	Stoe IPDS 2T	Stoe IPDS 2T
Absorption correction	Numerical *X-AREA* and *X-RED* (Stoe & Cie, 2002 ▸)	Numerical *X-AREA* and *X-RED* (Stoe & Cie, 2002 ▸)	Numerical *X-AREA* and *X-RED* (Stoe & Cie, 2002 ▸)	Numerical *X-AREA* and *X-RED* (Stoe & Cie, 2002 ▸)
*T*_min_, *T*_max_	0.851, 0.932	0.837, 0.888	0.838, 0.908	0.807, 0.938
No. of measured, independent and observed [*I* > 2σ(*I*)] reflections	5371, 2432, 2203	5377, 2495, 2239	18866, 7036, 6355	22018, 7124, 5922
*R*_int_	0.030	0.037	0.029	0.042
(sin θ/λ)_max_ (Å^−1^)	0.617	0.616	0.617	0.617

Refinement				
*R*[*F* ^2^ > 2σ(*F* ^2^)], *wR*(*F* ^2^), *S*	0.026, 0.054, 0.98	0.033, 0.074, 1.01	0.033, 0.075, 1.14	0.035, 0.083, 1.03
No. of reflections	2432	2495	7036	7124
No. of parameters	148	148	395	394
No. of restraints	2	2	2	2
H-atom treatment	H atoms treated by a mixture of independent and constrained refinement	H atoms treated by a mixture of independent and constrained refinement	H atoms treated by a mixture of independent and constrained refinement	H atoms treated by a mixture of independent and constrained refinement
Δρ_max_, Δρ_min_ (e Å^−3^)	0.17, −0.16	0.21, −0.42	0.40, −0.33	0.65, −0.36
Absolute structure	Flack *x* determined using 804 quotients [(*I* ^+^)−(*I* ^−^)]/[(*I* ^+^)+(*I* ^−^)] (Parsons *et al.*, 2013 ▸).	Flack *x* determined using 846 quotients [(*I* ^+^)−(*I* ^−^)]/[(*I* ^+^)+(*I* ^−^)] (Parsons *et al.*, 2013 ▸).	–	–
Absolute structure parameter	0.005 (17)	−0.03 (3)	–	–

Computer programs: *X-AREA* *X-AREA* and *X-RED* (Stoe & Cie, 2002 ▸), *SHELXT2014/5* (Sheldrick, 2015*a* ▸), *SHELXL2018/3* (Sheldrick, 2015*b* ▸), *DIAMOND* (Brandenburg, 1999 ▸) and *publCIF* (Westrip, 2010 ▸).

## supplementary crystallographic information

### Dichloridobis(3-cyclopropyl-N,N'-diisopropylprop-2-ynamidine)manganese(II) (3) Crystal data

**Table:**

[MnCl_2_(C_12_H_20_N_2_)_2_]	*D*_x_ = 1.221 Mg m^−^^3^
*M**_r_* = 510.44	Mo *K*α radiation, λ = 0.71073 Å
Orthorhombic, *F**d**d*2	Cell parameters from 5371 reflections
*a* = 17.6701 (10) Å	θ = 2.4–26.0°
*b* = 30.9809 (19) Å	µ = 0.69 mm^−^^1^
*c* = 10.1452 (5) Å	*T* = 153 K
*V* = 5553.8 (5) Å^3^	Plate, colorless
*Z* = 8	0.33 × 0.24 × 0.10 mm
*F*(000) = 2168	

### Dichloridobis(3-cyclopropyl-N,N'-diisopropylprop-2-ynamidine)manganese(II) (3) Data collection

**Table:**

Stoe IPDS 2T diffractometer	2432 independent reflections
Radiation source: fine-focus sealed tube	2203 reflections with *I* > 2σ(*I*)
Detector resolution: 6.67 pixels mm^-1^	*R*_int_ = 0.030
area detector scans	θ_max_ = 26.0°, θ_min_ = 2.4°
Absorption correction: numerical X-Area and X-Red (Stoe & Cie, 2002)	*h* = −21→19
*T*_min_ = 0.851, *T*_max_ = 0.932	*k* = −38→36
5371 measured reflections	*l* = −10→12

### Dichloridobis(3-cyclopropyl-N,N'-diisopropylprop-2-ynamidine)manganese(II) (3) Refinement

**Table:**

Refinement on *F*^2^	Hydrogen site location: mixed
Least-squares matrix: full	H atoms treated by a mixture of independent and constrained refinement
*R*[*F*^2^ > 2σ(*F*^2^)] = 0.026	*w* = 1/[σ^2^(*F*_o_^2^) + (0.0295*P*)^2^] where *P* = (*F*_o_^2^ + 2*F*_c_^2^)/3
*wR*(*F*^2^) = 0.054	(Δ/σ)_max_ < 0.001
*S* = 0.98	Δρ_max_ = 0.17 e Å^−^^3^
2432 reflections	Δρ_min_ = −0.16 e Å^−^^3^
148 parameters	Absolute structure: Flack *x* determined using 804 quotients [(*I*^+^)-(*I*^-^)]/[(*I*^+^)+(*I*^-^)] (Parsons *et al.*, 2013).
2 restraints	Absolute structure parameter: 0.005 (17)
Primary atom site location: dual	

### Dichloridobis(3-cyclopropyl-N,N'-diisopropylprop-2-ynamidine)manganese(II) (3) Special details

**Table:**

Geometry. All esds (except the esd in the dihedral angle between two l.s. planes) are estimated using the full covariance matrix. The cell esds are taken into account individually in the estimation of esds in distances, angles and torsion angles; correlations between esds in cell parameters are only used when they are defined by crystal symmetry. An approximate (isotropic) treatment of cell esds is used for estimating esds involving l.s. planes.

### Dichloridobis(3-cyclopropyl-N,N'-diisopropylprop-2-ynamidine)manganese(II) (3) Fractional atomic coordinates and isotropic or equivalent isotropic displacement parameters (Å^2^)

**Table:**

	*x*	*y*	*z*	*U*_iso_*/*U*_eq_	
C1	0.36882 (15)	0.54929 (8)	0.4904 (3)	0.0249 (6)	
C2	0.33550 (15)	0.58031 (8)	0.5793 (3)	0.0275 (6)	
C3	0.30913 (16)	0.60729 (9)	0.6494 (3)	0.0302 (7)	
C4	0.28074 (18)	0.63992 (9)	0.7356 (3)	0.0356 (7)	
H2	0.319053	0.653154	0.795855	0.043*	
C5	0.20206 (19)	0.63718 (12)	0.7899 (4)	0.0499 (9)	
H4	0.170588	0.612238	0.763079	0.060*	
H3	0.193744	0.647216	0.881390	0.060*	
C6	0.2193 (2)	0.66970 (11)	0.6900 (4)	0.0533 (10)	
H5	0.221801	0.700214	0.718620	0.064*	
H6	0.198644	0.665236	0.600306	0.064*	
C7	0.48948 (16)	0.58097 (9)	0.5311 (3)	0.0306 (7)	
H7	0.459936	0.594392	0.604475	0.037*	
C8	0.5092 (2)	0.61607 (9)	0.4321 (4)	0.0483 (9)	
H8	0.462550	0.629138	0.398310	0.072*	
H9	0.537769	0.603441	0.358799	0.072*	
H10	0.539979	0.638262	0.475250	0.072*	
C9	0.55984 (19)	0.56063 (11)	0.5888 (5)	0.0523 (10)	
H11	0.545423	0.536465	0.645823	0.078*	
H12	0.587472	0.582183	0.640576	0.078*	
H13	0.592242	0.550058	0.517331	0.078*	
C10	0.24079 (15)	0.51535 (9)	0.4664 (3)	0.0309 (6)	
H14	0.231615	0.528595	0.554861	0.037*	
C11	0.1891 (2)	0.53643 (15)	0.3699 (5)	0.0730 (14)	
H15	0.198631	0.567589	0.368767	0.110*	
H16	0.136417	0.531090	0.395577	0.110*	
H17	0.198092	0.524486	0.281836	0.110*	
C12	0.2262 (2)	0.46796 (10)	0.4771 (5)	0.0589 (11)	
H18	0.263622	0.454904	0.536161	0.088*	
H19	0.230123	0.454725	0.389606	0.088*	
H20	0.175270	0.463185	0.512437	0.088*	
Cl	0.40889 (4)	0.46957 (2)	0.20903 (8)	0.03750 (19)	
N1	0.44187 (12)	0.54779 (6)	0.4682 (2)	0.0244 (5)	
N2	0.32035 (14)	0.52226 (8)	0.4319 (3)	0.0329 (6)	
H1	0.3386 (17)	0.5071 (10)	0.370 (3)	0.039*	
Mn	0.500000	0.500000	0.35079 (6)	0.02229 (14)	

### Dichloridobis(3-cyclopropyl-N,N'-diisopropylprop-2-ynamidine)manganese(II) (3) Atomic displacement parameters (Å^2^)

**Table:**

	*U*^11^	*U*^22^	*U*^33^	*U*^12^	*U*^13^	*U*^23^
C1	0.0270 (13)	0.0210 (12)	0.0267 (16)	0.0043 (11)	0.0022 (12)	−0.0021 (10)
C2	0.0233 (13)	0.0260 (13)	0.0333 (18)	−0.0001 (11)	0.0014 (13)	−0.0054 (12)
C3	0.0241 (14)	0.0314 (14)	0.0350 (17)	−0.0011 (12)	0.0005 (13)	−0.0042 (12)
C4	0.0299 (15)	0.0393 (15)	0.038 (2)	0.0014 (12)	0.0019 (15)	−0.0162 (14)
C5	0.0372 (19)	0.056 (2)	0.057 (2)	−0.0006 (17)	0.0173 (18)	−0.0191 (17)
C6	0.054 (2)	0.052 (2)	0.055 (3)	0.0212 (17)	0.0009 (19)	−0.0145 (18)
C7	0.0229 (15)	0.0293 (14)	0.0396 (18)	0.0019 (12)	0.0027 (13)	−0.0124 (12)
C8	0.045 (2)	0.0315 (15)	0.068 (3)	−0.0095 (14)	0.0084 (19)	−0.0053 (15)
C9	0.0413 (17)	0.0463 (17)	0.069 (3)	0.0074 (15)	−0.020 (2)	−0.024 (2)
C10	0.0228 (14)	0.0325 (13)	0.0372 (18)	0.0001 (11)	0.0046 (15)	−0.0029 (12)
C11	0.0348 (18)	0.099 (3)	0.085 (4)	0.014 (2)	0.005 (2)	0.044 (3)
C12	0.0396 (18)	0.0394 (17)	0.098 (4)	−0.0071 (15)	0.008 (2)	0.005 (2)
Cl	0.0345 (4)	0.0426 (4)	0.0354 (4)	0.0088 (3)	−0.0083 (4)	−0.0170 (3)
N1	0.0231 (11)	0.0225 (10)	0.0276 (14)	0.0023 (8)	0.0001 (11)	−0.0048 (10)
N2	0.0239 (12)	0.0346 (12)	0.0402 (17)	−0.0013 (10)	0.0060 (11)	−0.0183 (11)
Mn	0.0229 (3)	0.0206 (2)	0.0234 (3)	0.0048 (3)	0.000	0.000

### Dichloridobis(3-cyclopropyl-N,N'-diisopropylprop-2-ynamidine)manganese(II) (3) Geometric parameters (Å, º)

**Table:**

C1—N1	1.311 (3)	C8—H9	0.9800
C1—N2	1.337 (4)	C8—H10	0.9800
C1—C2	1.444 (4)	C9—H11	0.9800
C2—C3	1.192 (4)	C9—H12	0.9800
C3—C4	1.428 (4)	C9—H13	0.9800
C4—C5	1.498 (4)	C10—N2	1.465 (3)
C4—C6	1.498 (5)	C10—C11	1.491 (5)
C4—H2	1.0000	C10—C12	1.494 (4)
C5—C6	1.461 (6)	C10—H14	1.0000
C5—H4	0.9900	C11—H15	0.9800
C5—H3	0.9900	C11—H16	0.9800
C6—H5	0.9900	C11—H17	0.9800
C6—H6	0.9900	C12—H18	0.9800
C7—N1	1.473 (3)	C12—H19	0.9800
C7—C9	1.512 (4)	C12—H20	0.9800
C7—C8	1.521 (5)	Cl—Mn	2.3556 (8)
C7—H7	1.0000	N1—Mn	2.160 (2)
C8—H8	0.9800	N2—H1	0.85 (2)
			
N1—C1—N2	122.2 (2)	C7—C9—H12	109.5
N1—C1—C2	122.2 (2)	H11—C9—H12	109.5
N2—C1—C2	115.7 (2)	C7—C9—H13	109.5
C3—C2—C1	177.2 (3)	H11—C9—H13	109.5
C2—C3—C4	177.5 (3)	H12—C9—H13	109.5
C3—C4—C5	120.7 (3)	N2—C10—C11	111.6 (3)
C3—C4—C6	120.1 (3)	N2—C10—C12	109.1 (2)
C5—C4—C6	58.4 (2)	C11—C10—C12	111.9 (3)
C3—C4—H2	115.3	N2—C10—H14	108.1
C5—C4—H2	115.3	C11—C10—H14	108.1
C6—C4—H2	115.3	C12—C10—H14	108.1
C6—C5—C4	60.8 (2)	C10—C11—H15	109.5
C6—C5—H4	117.7	C10—C11—H16	109.5
C4—C5—H4	117.7	H15—C11—H16	109.5
C6—C5—H3	117.7	C10—C11—H17	109.5
C4—C5—H3	117.7	H15—C11—H17	109.5
H4—C5—H3	114.8	H16—C11—H17	109.5
C5—C6—C4	60.8 (2)	C10—C12—H18	109.5
C5—C6—H5	117.7	C10—C12—H19	109.5
C4—C6—H5	117.7	H18—C12—H19	109.5
C5—C6—H6	117.7	C10—C12—H20	109.5
C4—C6—H6	117.7	H18—C12—H20	109.5
H5—C6—H6	114.8	H19—C12—H20	109.5
N1—C7—C9	110.3 (2)	C1—N1—C7	117.6 (2)
N1—C7—C8	110.1 (3)	C1—N1—Mn	125.95 (18)
C9—C7—C8	111.4 (3)	C7—N1—Mn	116.44 (16)
N1—C7—H7	108.3	C1—N2—C10	126.9 (2)
C9—C7—H7	108.3	C1—N2—H1	116 (2)
C8—C7—H7	108.3	C10—N2—H1	117 (2)
C7—C8—H8	109.5	N1—Mn—N1^i^	113.06 (13)
C7—C8—H9	109.5	N1—Mn—Cl	106.62 (6)
H8—C8—H9	109.5	N1^i^—Mn—Cl	112.79 (6)
C7—C8—H10	109.5	N1—Mn—Cl^i^	112.80 (6)
H8—C8—H10	109.5	N1^i^—Mn—Cl^i^	106.62 (6)
H9—C8—H10	109.5	Cl—Mn—Cl^i^	104.74 (5)
C7—C9—H11	109.5		
			
C3—C4—C5—C6	−108.6 (4)	C8—C7—N1—C1	−100.7 (3)
C3—C4—C6—C5	109.7 (3)	C9—C7—N1—Mn	−42.7 (3)
N2—C1—N1—C7	176.0 (3)	C8—C7—N1—Mn	80.7 (2)
C2—C1—N1—C7	−3.8 (4)	N1—C1—N2—C10	167.7 (3)
N2—C1—N1—Mn	−5.5 (4)	C2—C1—N2—C10	−12.5 (4)
C2—C1—N1—Mn	174.66 (19)	C11—C10—N2—C1	104.8 (4)
C9—C7—N1—C1	135.9 (3)	C12—C10—N2—C1	−131.2 (3)

Symmetry code: (i) −*x*+1, −*y*+1, *z*.

### Dichloridobis(3-cyclopropyl-N,N'-diisopropylprop-2-ynamidine)manganese(II) (3) Hydrogen-bond geometry (Å, º)

**Table:**

*D*—H···*A*	*D*—H	H···*A*	*D*···*A*	*D*—H···*A*
N2—H1···Cl	0.85 (2)	2.36 (2)	3.197 (3)	170 (3)
C8—H9···Cl^i^	0.98	2.88	3.776 (4)	152
C5—H4···Cl^ii^	0.99	2.95	3.931 (4)	172
C10—H14···Cl^ii^	1.00	2.93	3.643 (3)	129
C4—H2···Cl^iii^	1.00	2.67	3.516 (3)	143

Symmetry codes: (i) −*x*+1, −*y*+1, *z*; (ii) −*x*+1/2, −*y*+1, *z*+1/2; (iii) −*x*+3/4, *y*+1/4, *z*+3/4.

### Dichloridobis(3-cyclopropyl-N,N'-diisopropylprop-2-ynamidine)iron(II) (4) Crystal data

**Table:**

[FeCl_2_(C_12_H_20_N_2_)_2_]	*D*_x_ = 1.237 Mg m^−^^3^
*M**_r_* = 511.35	Mo *K*α radiation, λ = 0.71073 Å
Orthorhombic, *F**d**d*2	Cell parameters from 5377 reflections
*a* = 17.5703 (9) Å	θ = 2.4–26.0°
*b* = 30.9167 (12) Å	µ = 0.76 mm^−^^1^
*c* = 10.1110 (6) Å	*T* = 153 K
*V* = 5492.5 (5) Å^3^	Block, colorless
*Z* = 8	0.27 × 0.25 × 0.25 mm
*F*(000) = 2176	

### Dichloridobis(3-cyclopropyl-N,N'-diisopropylprop-2-ynamidine)iron(II) (4) Data collection

**Table:**

Stoe IPDS 2T diffractometer	2495 independent reflections
Radiation source: fine-focus sealed tube	2239 reflections with *I* > 2σ(*I*)
Detector resolution: 6.67 pixels mm^-1^	*R*_int_ = 0.037
area detector scans	θ_max_ = 26.0°, θ_min_ = 2.4°
Absorption correction: numerical X-Area and X-Red (Stoe & Cie, 2002)	*h* = −20→21
*T*_min_ = 0.837, *T*_max_ = 0.888	*k* = −38→36
5377 measured reflections	*l* = −10→12

### Dichloridobis(3-cyclopropyl-N,N'-diisopropylprop-2-ynamidine)iron(II) (4) Refinement

**Table:**

Refinement on *F*^2^	Hydrogen site location: mixed
Least-squares matrix: full	H atoms treated by a mixture of independent and constrained refinement
*R*[*F*^2^ > 2σ(*F*^2^)] = 0.033	*w* = 1/[σ^2^(*F*_o_^2^) + (0.045*P*)^2^] where *P* = (*F*_o_^2^ + 2*F*_c_^2^)/3
*wR*(*F*^2^) = 0.074	(Δ/σ)_max_ < 0.001
*S* = 1.01	Δρ_max_ = 0.21 e Å^−^^3^
2495 reflections	Δρ_min_ = −0.42 e Å^−^^3^
148 parameters	Absolute structure: Flack *x* determined using 846 quotients [(*I*^+^)-(*I*^-^)]/[(*I*^+^)+(*I*^-^)] (Parsons *et al.*, 2013).
2 restraints	Absolute structure parameter: −0.03 (3)
Primary atom site location: dual	

### Dichloridobis(3-cyclopropyl-N,N'-diisopropylprop-2-ynamidine)iron(II) (4) Special details

**Table:**

Geometry. All esds (except the esd in the dihedral angle between two l.s. planes) are estimated using the full covariance matrix. The cell esds are taken into account individually in the estimation of esds in distances, angles and torsion angles; correlations between esds in cell parameters are only used when they are defined by crystal symmetry. An approximate (isotropic) treatment of cell esds is used for estimating esds involving l.s. planes.

### Dichloridobis(3-cyclopropyl-N,N'-diisopropylprop-2-ynamidine)iron(II) (4) Fractional atomic coordinates and isotropic or equivalent isotropic displacement parameters (Å^2^)

**Table:**

	*x*	*y*	*z*	*U*_iso_*/*U*_eq_	
C1	0.3709 (2)	0.54820 (11)	0.4918 (4)	0.0256 (7)	
C2	0.3378 (2)	0.57953 (11)	0.5794 (4)	0.0295 (8)	
C3	0.3110 (2)	0.60671 (12)	0.6491 (4)	0.0311 (8)	
C4	0.2823 (2)	0.63943 (13)	0.7355 (4)	0.0370 (9)	
H2	0.320708	0.652558	0.796514	0.044*	
C5	0.2031 (3)	0.63654 (17)	0.7896 (5)	0.0516 (12)	
H4	0.171514	0.611617	0.761998	0.062*	
H3	0.194600	0.646416	0.881537	0.062*	
C6	0.2208 (3)	0.66945 (15)	0.6897 (5)	0.0506 (12)	
H5	0.223392	0.699970	0.718986	0.061*	
H6	0.200305	0.665170	0.599444	0.061*	
C7	0.4920 (2)	0.58015 (12)	0.5319 (4)	0.0309 (8)	
H7	0.462208	0.593479	0.605717	0.037*	
C8	0.5106 (3)	0.61521 (13)	0.4324 (6)	0.0506 (12)	
H8	0.463312	0.628650	0.401441	0.076*	
H9	0.537663	0.602488	0.357185	0.076*	
H10	0.542756	0.637176	0.474331	0.076*	
C9	0.5635 (2)	0.56028 (15)	0.5897 (6)	0.0531 (13)	
H11	0.549567	0.536538	0.649137	0.080*	
H12	0.591635	0.582334	0.639198	0.080*	
H13	0.595472	0.549128	0.517965	0.080*	
C10	0.24171 (19)	0.51490 (12)	0.4677 (4)	0.0313 (8)	
H14	0.232841	0.527648	0.557256	0.038*	
C11	0.1912 (3)	0.5375 (2)	0.3711 (7)	0.0717 (17)	
H15	0.204279	0.568265	0.368259	0.108*	
H16	0.137983	0.534183	0.398571	0.108*	
H17	0.198034	0.524779	0.283094	0.108*	
C12	0.2255 (3)	0.46742 (15)	0.4745 (7)	0.0597 (14)	
H18	0.261781	0.453556	0.534649	0.090*	
H19	0.230443	0.454738	0.386081	0.090*	
H20	0.173590	0.462874	0.507208	0.090*	
Cl	0.41255 (5)	0.47033 (3)	0.20952 (10)	0.0389 (3)	
N1	0.44457 (15)	0.54633 (9)	0.4699 (3)	0.0251 (6)	
N2	0.32222 (17)	0.52105 (11)	0.4330 (4)	0.0323 (7)	
H1	0.341 (2)	0.5058 (13)	0.369 (4)	0.039*	
Fe	0.500000	0.500000	0.35458 (7)	0.02332 (17)	

### Dichloridobis(3-cyclopropyl-N,N'-diisopropylprop-2-ynamidine)iron(II) (4) Atomic displacement parameters (Å^2^)

**Table:**

	*U*^11^	*U*^22^	*U*^33^	*U*^12^	*U*^13^	*U*^23^
C1	0.0283 (17)	0.0242 (16)	0.0243 (19)	0.0046 (15)	0.0015 (14)	−0.0018 (14)
C2	0.0241 (17)	0.0299 (18)	0.035 (2)	0.0018 (15)	−0.0011 (15)	−0.0040 (16)
C3	0.0289 (19)	0.0305 (19)	0.034 (2)	−0.0010 (17)	0.0009 (16)	−0.0044 (16)
C4	0.0326 (19)	0.040 (2)	0.038 (2)	0.0020 (17)	0.0027 (17)	−0.0109 (18)
C5	0.041 (2)	0.062 (3)	0.051 (3)	−0.001 (2)	0.016 (2)	−0.020 (2)
C6	0.051 (3)	0.053 (3)	0.047 (3)	0.021 (2)	0.001 (2)	−0.013 (2)
C7	0.0254 (19)	0.0298 (19)	0.038 (2)	0.0034 (16)	−0.0001 (16)	−0.0123 (15)
C8	0.048 (3)	0.033 (2)	0.071 (3)	−0.0063 (19)	0.011 (2)	−0.005 (2)
C9	0.041 (2)	0.051 (2)	0.067 (4)	0.008 (2)	−0.023 (2)	−0.024 (3)
C10	0.0236 (17)	0.0345 (18)	0.036 (2)	−0.0011 (15)	0.0012 (17)	−0.0033 (16)
C11	0.037 (2)	0.099 (4)	0.080 (4)	0.016 (3)	0.001 (3)	0.039 (4)
C12	0.039 (2)	0.043 (2)	0.097 (5)	−0.010 (2)	0.002 (3)	0.001 (3)
Cl	0.0359 (5)	0.0484 (5)	0.0324 (5)	0.0061 (4)	−0.0068 (4)	−0.0161 (4)
N1	0.0225 (13)	0.0273 (14)	0.0257 (16)	0.0020 (12)	0.0005 (12)	−0.0032 (13)
N2	0.0243 (15)	0.0359 (16)	0.037 (2)	−0.0002 (13)	0.0039 (13)	−0.0130 (13)
Fe	0.0243 (3)	0.0243 (3)	0.0214 (3)	0.0044 (3)	0.000	0.000

### Dichloridobis(3-cyclopropyl-N,N'-diisopropylprop-2-ynamidine)iron(II) (4) Geometric parameters (Å, º)

**Table:**

C1—N1	1.315 (4)	C8—H9	0.9800
C1—N2	1.337 (5)	C8—H10	0.9800
C1—C2	1.435 (5)	C9—H11	0.9800
C2—C3	1.193 (5)	C9—H12	0.9800
C3—C4	1.429 (5)	C9—H13	0.9800
C4—C5	1.497 (6)	C10—N2	1.470 (4)
C4—C6	1.498 (6)	C10—C11	1.493 (6)
C4—H2	1.0000	C10—C12	1.497 (6)
C5—C6	1.467 (7)	C10—H14	1.0000
C5—H4	0.9900	C11—H15	0.9800
C5—H3	0.9900	C11—H16	0.9800
C6—H5	0.9900	C11—H17	0.9800
C6—H6	0.9900	C12—H18	0.9800
C7—N1	1.477 (5)	C12—H19	0.9800
C7—C8	1.514 (6)	C12—H20	0.9800
C7—C9	1.515 (6)	Cl—Fe	2.3139 (10)
C7—H7	1.0000	N1—Fe	2.088 (3)
C8—H8	0.9800	N2—H1	0.87 (2)
			
N1—C1—N2	121.8 (3)	C7—C9—H12	109.5
N1—C1—C2	122.1 (3)	H11—C9—H12	109.5
N2—C1—C2	116.1 (3)	C7—C9—H13	109.5
C3—C2—C1	177.6 (4)	H11—C9—H13	109.5
C2—C3—C4	177.3 (4)	H12—C9—H13	109.5
C3—C4—C5	120.6 (4)	N2—C10—C11	110.8 (4)
C3—C4—C6	120.3 (4)	N2—C10—C12	108.7 (3)
C5—C4—C6	58.6 (3)	C11—C10—C12	112.0 (4)
C3—C4—H2	115.2	N2—C10—H14	108.4
C5—C4—H2	115.2	C11—C10—H14	108.4
C6—C4—H2	115.2	C12—C10—H14	108.4
C6—C5—C4	60.7 (3)	C10—C11—H15	109.5
C6—C5—H4	117.7	C10—C11—H16	109.5
C4—C5—H4	117.7	H15—C11—H16	109.5
C6—C5—H3	117.7	C10—C11—H17	109.5
C4—C5—H3	117.7	H15—C11—H17	109.5
H4—C5—H3	114.8	H16—C11—H17	109.5
C5—C6—C4	60.7 (3)	C10—C12—H18	109.5
C5—C6—H5	117.7	C10—C12—H19	109.5
C4—C6—H5	117.7	H18—C12—H19	109.5
C5—C6—H6	117.7	C10—C12—H20	109.5
C4—C6—H6	117.7	H18—C12—H20	109.5
H5—C6—H6	114.8	H19—C12—H20	109.5
N1—C7—C8	110.2 (3)	C1—N1—C7	116.9 (3)
N1—C7—C9	110.1 (3)	C1—N1—Fe	125.7 (2)
C8—C7—C9	111.6 (4)	C7—N1—Fe	117.4 (2)
N1—C7—H7	108.3	C1—N2—C10	126.2 (3)
C8—C7—H7	108.3	C1—N2—H1	115 (3)
C9—C7—H7	108.3	C10—N2—H1	118 (3)
C7—C8—H8	109.5	N1^i^—Fe—N1	112.13 (17)
C7—C8—H9	109.5	N1^i^—Fe—Cl	113.06 (9)
H8—C8—H9	109.5	N1—Fe—Cl	108.42 (8)
C7—C8—H10	109.5	N1^i^—Fe—Cl^i^	108.42 (8)
H8—C8—H10	109.5	N1—Fe—Cl^i^	113.06 (9)
H9—C8—H10	109.5	Cl—Fe—Cl^i^	101.32 (6)
C7—C9—H11	109.5		
			
C3—C4—C5—C6	−109.0 (5)	C9—C7—N1—C1	137.1 (4)
C3—C4—C6—C5	109.5 (4)	C8—C7—N1—Fe	80.4 (3)
N2—C1—N1—C7	175.2 (3)	C9—C7—N1—Fe	−43.2 (4)
C2—C1—N1—C7	−4.4 (5)	N1—C1—N2—C10	168.1 (4)
N2—C1—N1—Fe	−4.5 (5)	C2—C1—N2—C10	−12.3 (6)
C2—C1—N1—Fe	175.9 (3)	C11—C10—N2—C1	102.8 (5)
C8—C7—N1—C1	−99.4 (4)	C12—C10—N2—C1	−133.6 (5)

Symmetry code: (i) −*x*+1, −*y*+1, *z*.

### Dichloridobis(3-cyclopropyl-N,N'-diisopropylprop-2-ynamidine)iron(II) (4) Hydrogen-bond geometry (Å, º)

**Table:**

*D*—H···*A*	*D*—H	H···*A*	*D*···*A*	*D*—H···*A*
N2—H1···Cl	0.87 (2)	2.32 (3)	3.175 (3)	169 (4)
C8—H9···Cl^i^	0.98	2.84	3.728 (5)	151
C5—H4···Cl^ii^	0.99	2.98	3.963 (5)	172
C10—H14···Cl^ii^	1.00	2.98	3.679 (4)	128
C4—H2···Cl^iii^	1.00	2.68	3.510 (4)	140

Symmetry codes: (i) −*x*+1, −*y*+1, *z*; (ii) −*x*+1/2, −*y*+1, *z*+1/2; (iii) −*x*+3/4, *y*+1/4, *z*+3/4.

### Dichloridobis(N,N'-dicyclohexyl-3-cyclopropylprop-2-ynamidine)iron(II) (5) Crystal data

**Table:**

[FeCl_2_(C_18_H_28_N_2_)_2_]	*F*(000) = 1440
*M**_r_* = 671.59	*D*_x_ = 1.238 Mg m^−^^3^
Monoclinic, *P*2_1_/*c*	Mo *K*α radiation, λ = 0.71073 Å
*a* = 13.905 (7) Å	Cell parameters from 17648 reflections
*b* = 12.500 (6) Å	θ = 1.9–25.4°
*c* = 20.742 (11) Å	µ = 0.60 mm^−^^1^
β = 92.24 (4)°	*T* = 100 K
*V* = 3603 (3) Å^3^	Plate, colorless
*Z* = 4	0.26 × 0.19 × 0.12 mm

### Dichloridobis(N,N'-dicyclohexyl-3-cyclopropylprop-2-ynamidine)iron(II) (5) Data collection

**Table:**

Stoe IPDS 2T diffractometer	7036 independent reflections
Radiation source: fine-focus sealed tube	6355 reflections with *I* > 2σ(*I*)
Detector resolution: 6.67 pixels mm^-1^	*R*_int_ = 0.029
area detector scans	θ_max_ = 26.0°, θ_min_ = 1.9°
Absorption correction: numerical X-Area and X-Red (Stoe & Cie, 2002)	*h* = −17→16
*T*_min_ = 0.838, *T*_max_ = 0.908	*k* = −14→15
18866 measured reflections	*l* = −25→25

### Dichloridobis(N,N'-dicyclohexyl-3-cyclopropylprop-2-ynamidine)iron(II) (5) Refinement

**Table:**

Refinement on *F*^2^	Hydrogen site location: mixed
Least-squares matrix: full	H atoms treated by a mixture of independent and constrained refinement
*R*[*F*^2^ > 2σ(*F*^2^)] = 0.033	*w* = 1/[σ^2^(*F*_o_^2^) + (0.0293*P*)^2^ + 1.8416*P*] where *P* = (*F*_o_^2^ + 2*F*_c_^2^)/3
*wR*(*F*^2^) = 0.075	(Δ/σ)_max_ = 0.001
*S* = 1.14	Δρ_max_ = 0.40 e Å^−^^3^
7036 reflections	Δρ_min_ = −0.33 e Å^−^^3^
395 parameters	Extinction correction: SHELXL-2018/3 (Sheldrick 2015b), Fc^*^=kFc[1+0.001xFc^2^λ^3^/sin(2θ)]^-1/4^
2 restraints	Extinction coefficient: 0.0028 (4)
Primary atom site location: dual	

### Dichloridobis(N,N'-dicyclohexyl-3-cyclopropylprop-2-ynamidine)iron(II) (5) Special details

**Table:**

Geometry. All esds (except the esd in the dihedral angle between two l.s. planes) are estimated using the full covariance matrix. The cell esds are taken into account individually in the estimation of esds in distances, angles and torsion angles; correlations between esds in cell parameters are only used when they are defined by crystal symmetry. An approximate (isotropic) treatment of cell esds is used for estimating esds involving l.s. planes.

### Dichloridobis(N,N'-dicyclohexyl-3-cyclopropylprop-2-ynamidine)iron(II) (5) Fractional atomic coordinates and isotropic or equivalent isotropic displacement parameters (Å^2^)

**Table:**

	*x*	*y*	*z*	*U*_iso_*/*U*_eq_	
C1	0.18374 (11)	0.73239 (12)	0.51035 (7)	0.0161 (3)	
C2	0.18720 (11)	0.66376 (13)	0.45462 (7)	0.0168 (3)	
C3	0.19066 (11)	0.60813 (13)	0.40810 (7)	0.0170 (3)	
C4	0.19602 (12)	0.54251 (13)	0.35178 (8)	0.0177 (3)	
H2	0.139809	0.546681	0.320304	0.021*	
C5	0.24578 (12)	0.43512 (13)	0.35720 (8)	0.0212 (3)	
H3	0.273324	0.413811	0.400026	0.025*	
H4	0.219025	0.375546	0.330658	0.025*	
C6	0.29280 (13)	0.52459 (14)	0.32327 (8)	0.0244 (4)	
H5	0.295258	0.520379	0.275701	0.029*	
H6	0.349545	0.558635	0.345053	0.029*	
C7	0.34866 (11)	0.69174 (12)	0.53244 (7)	0.0155 (3)	
H7	0.331881	0.624187	0.508755	0.019*	
C8	0.40490 (12)	0.76358 (13)	0.48786 (8)	0.0194 (3)	
H8	0.420275	0.831957	0.510038	0.023*	
H9	0.364745	0.779817	0.448615	0.023*	
C9	0.49799 (12)	0.70959 (15)	0.46867 (8)	0.0231 (4)	
H10	0.482171	0.646833	0.441134	0.028*	
H11	0.535678	0.760259	0.443040	0.028*	
C10	0.55896 (12)	0.67335 (16)	0.52738 (8)	0.0261 (4)	
H12	0.614416	0.631283	0.512864	0.031*	
H13	0.584414	0.736967	0.550800	0.031*	
C11	0.50120 (12)	0.60571 (14)	0.57284 (8)	0.0214 (3)	
H14	0.541338	0.589208	0.612053	0.026*	
H15	0.483612	0.537232	0.551501	0.026*	
C12	0.41006 (11)	0.66312 (13)	0.59209 (7)	0.0173 (3)	
H16	0.372995	0.616336	0.620556	0.021*	
H17	0.427449	0.729027	0.616312	0.021*	
C13	0.01049 (11)	0.75831 (13)	0.48221 (7)	0.0171 (3)	
H18	0.026349	0.739901	0.436925	0.020*	
C14	−0.05565 (12)	0.85514 (13)	0.47973 (8)	0.0196 (3)	
H19	−0.023073	0.915540	0.458747	0.024*	
H20	−0.070252	0.877139	0.524145	0.024*	
C15	−0.14923 (12)	0.82858 (14)	0.44199 (8)	0.0210 (3)	
H21	−0.193205	0.890760	0.443257	0.025*	
H22	−0.135037	0.814825	0.396327	0.025*	
C16	−0.19864 (12)	0.73113 (15)	0.46954 (8)	0.0234 (4)	
H23	−0.256468	0.713587	0.442226	0.028*	
H24	−0.219839	0.747881	0.513424	0.028*	
C17	−0.13164 (13)	0.63472 (14)	0.47271 (9)	0.0246 (4)	
H25	−0.116038	0.612820	0.428457	0.030*	
H26	−0.164296	0.574063	0.493383	0.030*	
C18	−0.03899 (12)	0.66161 (13)	0.51117 (8)	0.0225 (3)	
H27	−0.054045	0.677124	0.556482	0.027*	
H28	0.004969	0.599326	0.511027	0.027*	
C19	0.34950 (11)	0.72475 (12)	0.75081 (7)	0.0143 (3)	
C20	0.35781 (11)	0.64968 (12)	0.80354 (7)	0.0165 (3)	
C21	0.35957 (11)	0.58263 (13)	0.84443 (7)	0.0179 (3)	
C22	0.35866 (13)	0.50039 (14)	0.89235 (8)	0.0246 (4)	
H30	0.422855	0.471409	0.907068	0.030*	
C23	0.28235 (16)	0.50218 (17)	0.94202 (9)	0.0337 (4)	
H31	0.300638	0.477198	0.986107	0.040*	
H32	0.235451	0.561790	0.940136	0.040*	
C24	0.27579 (14)	0.42104 (15)	0.88987 (9)	0.0289 (4)	
H33	0.225023	0.430424	0.855535	0.035*	
H34	0.290246	0.345786	0.901532	0.035*	
C25	0.19047 (11)	0.65995 (12)	0.72937 (7)	0.0161 (3)	
H35	0.217077	0.593327	0.749938	0.019*	
C26	0.12855 (12)	0.71616 (14)	0.77799 (8)	0.0212 (3)	
H36	0.167993	0.731520	0.817682	0.025*	
H37	0.105625	0.785192	0.759745	0.025*	
C27	0.04233 (13)	0.64810 (16)	0.79503 (8)	0.0278 (4)	
H38	0.001660	0.688525	0.824667	0.033*	
H39	0.065054	0.582447	0.817556	0.033*	
C28	−0.01730 (12)	0.61746 (16)	0.73466 (8)	0.0263 (4)	
H40	−0.071153	0.570605	0.746631	0.032*	
H41	−0.044864	0.682696	0.714150	0.032*	
C29	0.04498 (12)	0.55937 (14)	0.68713 (8)	0.0226 (4)	
H42	0.005834	0.541935	0.647639	0.027*	
H43	0.068426	0.491401	0.706556	0.027*	
C30	0.13082 (11)	0.62796 (13)	0.66927 (7)	0.0173 (3)	
H44	0.171569	0.587352	0.639778	0.021*	
H45	0.107523	0.693073	0.646411	0.021*	
C31	0.51602 (11)	0.78749 (12)	0.78087 (7)	0.0154 (3)	
H46	0.501379	0.770673	0.826590	0.018*	
C32	0.55969 (12)	0.89931 (13)	0.77874 (8)	0.0198 (3)	
H47	0.514586	0.951259	0.797183	0.024*	
H48	0.569124	0.919683	0.733302	0.024*	
C33	0.65602 (12)	0.90446 (13)	0.81646 (8)	0.0212 (3)	
H49	0.684691	0.976251	0.811104	0.025*	
H50	0.645231	0.893817	0.862918	0.025*	
C34	0.72569 (12)	0.82016 (14)	0.79387 (8)	0.0220 (3)	
H51	0.785401	0.822357	0.821440	0.026*	
H52	0.742891	0.835846	0.748973	0.026*	
C35	0.68131 (12)	0.70902 (14)	0.79694 (8)	0.0223 (4)	
H53	0.669835	0.690603	0.842419	0.027*	
H54	0.726701	0.655847	0.780054	0.027*	
C36	0.58615 (12)	0.70403 (13)	0.75741 (8)	0.0211 (3)	
H55	0.598301	0.716785	0.711324	0.025*	
H56	0.557596	0.631888	0.761341	0.025*	
N1	0.25819 (9)	0.74443 (10)	0.55028 (6)	0.0152 (3)	
N2	0.10047 (10)	0.78421 (11)	0.51761 (7)	0.0183 (3)	
H1	0.0964 (14)	0.8248 (14)	0.5499 (8)	0.022*	
N3	0.27221 (9)	0.72905 (10)	0.71271 (6)	0.0152 (3)	
N4	0.42557 (10)	0.78865 (11)	0.74247 (6)	0.0164 (3)	
H29	0.4208 (14)	0.8347 (14)	0.7126 (8)	0.020*	
Fe	0.25601 (2)	0.83290 (2)	0.63472 (2)	0.01437 (8)	
Cl1	0.37746 (3)	0.95842 (3)	0.62727 (2)	0.02006 (10)	
Cl2	0.11601 (3)	0.92675 (3)	0.64870 (2)	0.02200 (10)	

### Dichloridobis(N,N'-dicyclohexyl-3-cyclopropylprop-2-ynamidine)iron(II) (5) Atomic displacement parameters (Å^2^)

**Table:**

	*U*^11^	*U*^22^	*U*^33^	*U*^12^	*U*^13^	*U*^23^
C1	0.0192 (8)	0.0150 (7)	0.0144 (7)	−0.0004 (6)	0.0018 (6)	0.0015 (6)
C2	0.0155 (7)	0.0178 (8)	0.0171 (8)	0.0012 (6)	0.0000 (6)	0.0017 (6)
C3	0.0161 (7)	0.0175 (8)	0.0172 (8)	0.0000 (6)	−0.0004 (6)	0.0018 (6)
C4	0.0191 (8)	0.0179 (8)	0.0159 (7)	0.0001 (6)	−0.0013 (6)	−0.0025 (6)
C5	0.0242 (8)	0.0186 (8)	0.0209 (8)	0.0027 (7)	0.0011 (7)	−0.0009 (6)
C6	0.0272 (9)	0.0245 (9)	0.0218 (8)	0.0012 (7)	0.0064 (7)	−0.0033 (7)
C7	0.0165 (7)	0.0159 (7)	0.0140 (7)	0.0021 (6)	0.0020 (6)	−0.0008 (6)
C8	0.0212 (8)	0.0210 (8)	0.0163 (8)	0.0007 (7)	0.0034 (6)	0.0032 (6)
C9	0.0222 (8)	0.0286 (9)	0.0188 (8)	0.0022 (7)	0.0062 (7)	0.0037 (7)
C10	0.0164 (8)	0.0380 (10)	0.0240 (9)	0.0031 (7)	0.0026 (7)	0.0010 (8)
C11	0.0214 (8)	0.0267 (9)	0.0161 (8)	0.0074 (7)	0.0001 (6)	0.0011 (7)
C12	0.0186 (8)	0.0191 (8)	0.0142 (7)	0.0023 (6)	0.0015 (6)	0.0009 (6)
C13	0.0162 (8)	0.0187 (8)	0.0162 (7)	0.0004 (6)	−0.0005 (6)	−0.0011 (6)
C14	0.0190 (8)	0.0174 (8)	0.0223 (8)	0.0017 (6)	−0.0003 (6)	−0.0005 (6)
C15	0.0168 (8)	0.0241 (8)	0.0220 (8)	0.0028 (7)	−0.0002 (6)	−0.0005 (7)
C16	0.0176 (8)	0.0324 (10)	0.0201 (8)	−0.0038 (7)	0.0013 (6)	−0.0010 (7)
C17	0.0259 (9)	0.0227 (9)	0.0252 (9)	−0.0072 (7)	0.0003 (7)	0.0012 (7)
C18	0.0248 (8)	0.0197 (8)	0.0229 (8)	0.0007 (7)	−0.0013 (7)	0.0031 (7)
C19	0.0177 (7)	0.0127 (7)	0.0125 (7)	0.0012 (6)	0.0028 (6)	−0.0017 (6)
C20	0.0162 (7)	0.0178 (8)	0.0154 (7)	−0.0016 (6)	0.0004 (6)	−0.0014 (6)
C21	0.0180 (8)	0.0205 (8)	0.0153 (7)	−0.0008 (6)	0.0019 (6)	−0.0019 (6)
C22	0.0275 (9)	0.0249 (9)	0.0213 (8)	−0.0021 (7)	0.0000 (7)	0.0084 (7)
C23	0.0471 (12)	0.0335 (10)	0.0215 (9)	−0.0152 (9)	0.0115 (8)	0.0009 (8)
C24	0.0387 (10)	0.0209 (9)	0.0272 (9)	−0.0069 (8)	0.0034 (8)	0.0049 (7)
C25	0.0173 (7)	0.0163 (7)	0.0147 (7)	−0.0029 (6)	0.0016 (6)	0.0001 (6)
C26	0.0208 (8)	0.0270 (9)	0.0161 (8)	−0.0055 (7)	0.0036 (6)	−0.0053 (7)
C27	0.0257 (9)	0.0401 (11)	0.0180 (8)	−0.0120 (8)	0.0067 (7)	−0.0055 (7)
C28	0.0200 (8)	0.0377 (10)	0.0215 (8)	−0.0109 (8)	0.0040 (7)	−0.0023 (7)
C29	0.0246 (9)	0.0267 (9)	0.0164 (8)	−0.0093 (7)	0.0002 (7)	−0.0010 (7)
C30	0.0196 (8)	0.0186 (8)	0.0138 (7)	−0.0028 (6)	0.0021 (6)	−0.0018 (6)
C31	0.0164 (7)	0.0163 (7)	0.0134 (7)	−0.0024 (6)	−0.0002 (6)	−0.0001 (6)
C32	0.0197 (8)	0.0163 (8)	0.0232 (8)	−0.0018 (6)	−0.0007 (6)	0.0011 (6)
C33	0.0207 (8)	0.0182 (8)	0.0244 (8)	−0.0044 (7)	−0.0025 (7)	0.0009 (7)
C34	0.0179 (8)	0.0270 (9)	0.0210 (8)	−0.0029 (7)	−0.0012 (6)	0.0022 (7)
C35	0.0208 (8)	0.0210 (8)	0.0247 (8)	0.0039 (7)	−0.0025 (7)	−0.0027 (7)
C36	0.0213 (8)	0.0196 (8)	0.0221 (8)	0.0007 (7)	−0.0023 (7)	−0.0042 (7)
N1	0.0154 (6)	0.0160 (6)	0.0141 (6)	0.0015 (5)	0.0010 (5)	0.0004 (5)
N2	0.0179 (7)	0.0204 (7)	0.0166 (7)	0.0026 (6)	−0.0017 (5)	−0.0047 (5)
N3	0.0168 (6)	0.0157 (6)	0.0133 (6)	−0.0012 (5)	0.0020 (5)	−0.0011 (5)
N4	0.0179 (7)	0.0172 (7)	0.0140 (6)	−0.0023 (5)	−0.0006 (5)	0.0040 (5)
Fe	0.01670 (12)	0.01337 (12)	0.01306 (12)	0.00048 (9)	0.00100 (8)	−0.00022 (8)
Cl1	0.0227 (2)	0.01594 (18)	0.02139 (19)	−0.00324 (15)	−0.00072 (15)	0.00174 (14)
Cl2	0.0226 (2)	0.0198 (2)	0.0237 (2)	0.00596 (15)	0.00141 (15)	−0.00294 (15)

### Dichloridobis(N,N'-dicyclohexyl-3-cyclopropylprop-2-ynamidine)iron(II) (5) Geometric parameters (Å, º)

**Table:**

C1—N1	1.309 (2)	C20—C21	1.192 (2)
C1—N2	1.340 (2)	C21—C22	1.430 (2)
C1—C2	1.442 (2)	C22—C23	1.508 (3)
C2—C3	1.192 (2)	C22—C24	1.520 (3)
C3—C4	1.432 (2)	C22—H30	1.0000
C4—C6	1.508 (2)	C23—C24	1.483 (3)
C4—C5	1.512 (2)	C23—H31	0.9900
C4—H2	1.0000	C23—H32	0.9900
C5—C6	1.486 (2)	C24—H33	0.9900
C5—H3	0.9900	C24—H34	0.9900
C5—H4	0.9900	C25—N3	1.479 (2)
C6—H5	0.9900	C25—C26	1.523 (2)
C6—H6	0.9900	C25—C30	1.524 (2)
C7—N1	1.480 (2)	C25—H35	1.0000
C7—C12	1.518 (2)	C26—C27	1.523 (2)
C7—C8	1.526 (2)	C26—H36	0.9900
C7—H7	1.0000	C26—H37	0.9900
C8—C9	1.526 (2)	C27—C28	1.524 (2)
C8—H8	0.9900	C27—H38	0.9900
C8—H9	0.9900	C27—H39	0.9900
C9—C10	1.525 (2)	C28—C29	1.522 (3)
C9—H10	0.9900	C28—H40	0.9900
C9—H11	0.9900	C28—H41	0.9900
C10—C11	1.519 (2)	C29—C30	1.527 (2)
C10—H12	0.9900	C29—H42	0.9900
C10—H13	0.9900	C29—H43	0.9900
C11—C12	1.523 (2)	C30—H44	0.9900
C11—H14	0.9900	C30—H45	0.9900
C11—H15	0.9900	C31—N4	1.462 (2)
C12—H16	0.9900	C31—C36	1.521 (2)
C12—H17	0.9900	C31—C32	1.525 (2)
C13—N2	1.462 (2)	C31—H46	1.0000
C13—C14	1.520 (2)	C32—C33	1.526 (2)
C13—C18	1.526 (2)	C32—H47	0.9900
C13—H18	1.0000	C32—H48	0.9900
C14—C15	1.529 (2)	C33—C34	1.518 (2)
C14—H19	0.9900	C33—H49	0.9900
C14—H20	0.9900	C33—H50	0.9900
C15—C16	1.521 (2)	C34—C35	1.522 (2)
C15—H21	0.9900	C34—H51	0.9900
C15—H22	0.9900	C34—H52	0.9900
C16—C17	1.523 (3)	C35—C36	1.531 (2)
C16—H23	0.9900	C35—H53	0.9900
C16—H24	0.9900	C35—H54	0.9900
C17—C18	1.526 (2)	C36—H55	0.9900
C17—H25	0.9900	C36—H56	0.9900
C17—H26	0.9900	N1—Fe	2.0727 (15)
C18—H27	0.9900	N2—H1	0.844 (15)
C18—H28	0.9900	N3—Fe	2.0795 (15)
C19—N3	1.310 (2)	N4—H29	0.847 (15)
C19—N4	1.342 (2)	Fe—Cl2	2.3009 (10)
C19—C20	1.442 (2)	Fe—Cl1	2.3147 (9)
			
N1—C1—N2	122.59 (15)	C23—C22—H30	116.1
N1—C1—C2	121.70 (15)	C24—C22—H30	116.1
N2—C1—C2	115.70 (14)	C24—C23—C22	61.07 (13)
C3—C2—C1	179.10 (18)	C24—C23—H31	117.7
C2—C3—C4	179.02 (18)	C22—C23—H31	117.7
C3—C4—C6	118.74 (14)	C24—C23—H32	117.7
C3—C4—C5	119.09 (14)	C22—C23—H32	117.7
C6—C4—C5	58.96 (11)	H31—C23—H32	114.8
C3—C4—H2	116.0	C23—C24—C22	60.26 (13)
C6—C4—H2	116.0	C23—C24—H33	117.7
C5—C4—H2	116.0	C22—C24—H33	117.7
C6—C5—C4	60.36 (11)	C23—C24—H34	117.7
C6—C5—H3	117.7	C22—C24—H34	117.7
C4—C5—H3	117.7	H33—C24—H34	114.9
C6—C5—H4	117.7	N3—C25—C26	110.16 (13)
C4—C5—H4	117.7	N3—C25—C30	111.15 (13)
H3—C5—H4	114.9	C26—C25—C30	111.04 (13)
C5—C6—C4	60.67 (11)	N3—C25—H35	108.1
C5—C6—H5	117.7	C26—C25—H35	108.1
C4—C6—H5	117.7	C30—C25—H35	108.1
C5—C6—H6	117.7	C27—C26—C25	111.50 (14)
C4—C6—H6	117.7	C27—C26—H36	109.3
H5—C6—H6	114.8	C25—C26—H36	109.3
N1—C7—C12	110.96 (13)	C27—C26—H37	109.3
N1—C7—C8	110.50 (13)	C25—C26—H37	109.3
C12—C7—C8	110.32 (13)	H36—C26—H37	108.0
N1—C7—H7	108.3	C26—C27—C28	110.96 (14)
C12—C7—H7	108.3	C26—C27—H38	109.4
C8—C7—H7	108.3	C28—C27—H38	109.4
C7—C8—C9	110.96 (14)	C26—C27—H39	109.4
C7—C8—H8	109.4	C28—C27—H39	109.4
C9—C8—H8	109.4	H38—C27—H39	108.0
C7—C8—H9	109.4	C29—C28—C27	110.32 (15)
C9—C8—H9	109.4	C29—C28—H40	109.6
H8—C8—H9	108.0	C27—C28—H40	109.6
C10—C9—C8	111.94 (14)	C29—C28—H41	109.6
C10—C9—H10	109.2	C27—C28—H41	109.6
C8—C9—H10	109.2	H40—C28—H41	108.1
C10—C9—H11	109.2	C28—C29—C30	111.05 (14)
C8—C9—H11	109.2	C28—C29—H42	109.4
H10—C9—H11	107.9	C30—C29—H42	109.4
C11—C10—C9	111.72 (14)	C28—C29—H43	109.4
C11—C10—H12	109.3	C30—C29—H43	109.4
C9—C10—H12	109.3	H42—C29—H43	108.0
C11—C10—H13	109.3	C25—C30—C29	110.77 (13)
C9—C10—H13	109.3	C25—C30—H44	109.5
H12—C10—H13	107.9	C29—C30—H44	109.5
C10—C11—C12	111.46 (14)	C25—C30—H45	109.5
C10—C11—H14	109.3	C29—C30—H45	109.5
C12—C11—H14	109.3	H44—C30—H45	108.1
C10—C11—H15	109.3	N4—C31—C36	112.40 (13)
C12—C11—H15	109.3	N4—C31—C32	108.07 (13)
H14—C11—H15	108.0	C36—C31—C32	111.00 (14)
C7—C12—C11	110.16 (13)	N4—C31—H46	108.4
C7—C12—H16	109.6	C36—C31—H46	108.4
C11—C12—H16	109.6	C32—C31—H46	108.4
C7—C12—H17	109.6	C31—C32—C33	111.49 (13)
C11—C12—H17	109.6	C31—C32—H47	109.3
H16—C12—H17	108.1	C33—C32—H47	109.3
N2—C13—C14	110.21 (13)	C31—C32—H48	109.3
N2—C13—C18	111.59 (13)	C33—C32—H48	109.3
C14—C13—C18	111.26 (14)	H47—C32—H48	108.0
N2—C13—H18	107.9	C34—C33—C32	111.75 (14)
C14—C13—H18	107.9	C34—C33—H49	109.3
C18—C13—H18	107.9	C32—C33—H49	109.3
C13—C14—C15	110.26 (13)	C34—C33—H50	109.3
C13—C14—H19	109.6	C32—C33—H50	109.3
C15—C14—H19	109.6	H49—C33—H50	107.9
C13—C14—H20	109.6	C33—C34—C35	110.85 (14)
C15—C14—H20	109.6	C33—C34—H51	109.5
H19—C14—H20	108.1	C35—C34—H51	109.5
C16—C15—C14	111.69 (14)	C33—C34—H52	109.5
C16—C15—H21	109.3	C35—C34—H52	109.5
C14—C15—H21	109.3	H51—C34—H52	108.1
C16—C15—H22	109.3	C34—C35—C36	110.99 (14)
C14—C15—H22	109.3	C34—C35—H53	109.4
H21—C15—H22	107.9	C36—C35—H53	109.4
C15—C16—C17	111.43 (14)	C34—C35—H54	109.4
C15—C16—H23	109.3	C36—C35—H54	109.4
C17—C16—H23	109.3	H53—C35—H54	108.0
C15—C16—H24	109.3	C31—C36—C35	110.63 (13)
C17—C16—H24	109.3	C31—C36—H55	109.5
H23—C16—H24	108.0	C35—C36—H55	109.5
C16—C17—C18	110.53 (14)	C31—C36—H56	109.5
C16—C17—H25	109.5	C35—C36—H56	109.5
C18—C17—H25	109.5	H55—C36—H56	108.1
C16—C17—H26	109.5	C1—N1—C7	116.72 (13)
C18—C17—H26	109.5	C1—N1—Fe	123.97 (11)
H25—C17—H26	108.1	C7—N1—Fe	119.30 (10)
C13—C18—C17	110.69 (14)	C1—N2—C13	124.24 (14)
C13—C18—H27	109.5	C1—N2—H1	117.7 (14)
C17—C18—H27	109.5	C13—N2—H1	116.7 (14)
C13—C18—H28	109.5	C19—N3—C25	117.06 (13)
C17—C18—H28	109.5	C19—N3—Fe	123.80 (11)
H27—C18—H28	108.1	C25—N3—Fe	119.04 (10)
N3—C19—N4	122.01 (14)	C19—N4—C31	126.11 (13)
N3—C19—C20	121.58 (14)	C19—N4—H29	117.3 (13)
N4—C19—C20	116.41 (14)	C31—N4—H29	116.6 (13)
C21—C20—C19	174.78 (17)	N1—Fe—N3	108.64 (6)
C20—C21—C22	177.90 (18)	N1—Fe—Cl2	114.76 (5)
C21—C22—C23	119.33 (16)	N3—Fe—Cl2	106.66 (5)
C21—C22—C24	118.22 (15)	N1—Fe—Cl1	105.69 (5)
C23—C22—C24	58.66 (12)	N3—Fe—Cl1	114.71 (5)
C21—C22—H30	116.1	Cl2—Fe—Cl1	106.61 (4)
			
C3—C4—C5—C6	−107.90 (17)	C31—C32—C33—C34	54.38 (19)
C3—C4—C6—C5	108.49 (17)	C32—C33—C34—C35	−54.99 (19)
N1—C7—C8—C9	−179.50 (13)	C33—C34—C35—C36	56.42 (19)
C12—C7—C8—C9	57.43 (17)	N4—C31—C36—C35	177.45 (13)
C7—C8—C9—C10	−53.92 (19)	C32—C31—C36—C35	56.28 (18)
C8—C9—C10—C11	52.2 (2)	C34—C35—C36—C31	−57.25 (19)
C9—C10—C11—C12	−54.00 (19)	N2—C1—N1—C7	−173.40 (14)
N1—C7—C12—C11	178.12 (13)	C2—C1—N1—C7	5.2 (2)
C8—C7—C12—C11	−59.07 (18)	N2—C1—N1—Fe	5.9 (2)
C10—C11—C12—C7	57.54 (18)	C2—C1—N1—Fe	−175.49 (11)
N2—C13—C14—C15	179.28 (13)	C12—C7—N1—C1	−151.55 (14)
C18—C13—C14—C15	−56.38 (18)	C8—C7—N1—C1	85.76 (17)
C13—C14—C15—C16	55.25 (19)	C12—C7—N1—Fe	29.09 (16)
C14—C15—C16—C17	−55.31 (19)	C8—C7—N1—Fe	−93.61 (13)
C15—C16—C17—C18	55.58 (19)	N1—C1—N2—C13	−167.61 (15)
N2—C13—C18—C17	−178.89 (14)	C2—C1—N2—C13	13.7 (2)
C14—C13—C18—C17	57.55 (18)	C14—C13—N2—C1	−156.41 (15)
C16—C17—C18—C13	−56.52 (19)	C18—C13—N2—C1	79.44 (19)
C21—C22—C23—C24	107.03 (19)	N4—C19—N3—C25	−175.90 (14)
C21—C22—C24—C23	−108.9 (2)	C20—C19—N3—C25	5.0 (2)
N3—C25—C26—C27	178.69 (13)	N4—C19—N3—Fe	0.3 (2)
C30—C25—C26—C27	55.11 (18)	C20—C19—N3—Fe	−178.76 (11)
C25—C26—C27—C28	−55.9 (2)	C26—C25—N3—C19	85.50 (17)
C26—C27—C28—C29	56.7 (2)	C30—C25—N3—C19	−150.99 (14)
C27—C28—C29—C30	−57.4 (2)	C26—C25—N3—Fe	−90.90 (14)
N3—C25—C30—C29	−178.35 (13)	C30—C25—N3—Fe	32.62 (16)
C26—C25—C30—C29	−55.34 (18)	N3—C19—N4—C31	−177.79 (14)
C28—C29—C30—C25	56.85 (19)	C20—C19—N4—C31	1.3 (2)
N4—C31—C32—C33	−178.60 (13)	C36—C31—N4—C19	84.39 (19)
C36—C31—C32—C33	−54.92 (18)	C32—C31—N4—C19	−152.79 (15)

### Dichloridobis(N,N'-dicyclohexyl-3-cyclopropylprop-2-ynamidine)iron(II) (5) Hydrogen-bond geometry (Å, º)

**Table:**

*D*—H···*A*	*D*—H	H···*A*	*D*···*A*	*D*—H···*A*
N2—H1···Cl2	0.84 (2)	2.42 (2)	3.2511 (19)	169 (2)
N4—H29···Cl1	0.85 (2)	2.41 (2)	3.2459 (18)	170 (2)
C22—H30···Cl1^i^	1.00	2.90	3.744 (3)	143
C35—H53···Cl1^i^	0.99	3.05	3.613 (2)	118
C28—H40···Cl2^ii^	0.99	2.91	3.699 (2)	138

Symmetry codes: (i) −*x*+1, *y*−1/2, −*z*+3/2; (ii) −*x*, *y*−1/2, −*z*+3/2.

### Dichloridobis(N,N'-dicyclohexyl-3-cyclopropylprop-2-ynamidine)cobalt(II) (6) Crystal data

**Table:**

[CoCl_2_(C_18_H_28_N_2_)_2_]	*F*(000) = 1444
*M**_r_* = 674.67	*D*_x_ = 1.233 Mg m^−^^3^
Monoclinic, *P*2_1_/*c*	Mo *K*α radiation, λ = 0.71073 Å
*a* = 13.8898 (3) Å	Cell parameters from 25049 reflections
*b* = 12.5574 (3) Å	θ = 1.9–27.3°
*c* = 20.8394 (5) Å	µ = 0.65 mm^−^^1^
β = 91.717 (2)°	*T* = 153 K
*V* = 3633.17 (15) Å^3^	Rod, blue
*Z* = 4	0.39 × 0.19 × 0.10 mm

### Dichloridobis(N,N'-dicyclohexyl-3-cyclopropylprop-2-ynamidine)cobalt(II) (6) Data collection

**Table:**

Stoe IPDS 2T diffractometer	7124 independent reflections
Radiation source: fine-focus sealed tube	5922 reflections with *I* > 2σ(*I*)
Detector resolution: 6.67 pixels mm^-1^	*R*_int_ = 0.042
area detector scans	θ_max_ = 26.0°, θ_min_ = 2.2°
Absorption correction: numerical X-Area and X-Red (Stoe & Cie, 2002)	*h* = −15→17
*T*_min_ = 0.807, *T*_max_ = 0.938	*k* = −15→15
22018 measured reflections	*l* = −25→25

### Dichloridobis(N,N'-dicyclohexyl-3-cyclopropylprop-2-ynamidine)cobalt(II) (6) Refinement

**Table:**

Refinement on *F*^2^	Primary atom site location: dual
Least-squares matrix: full	Hydrogen site location: mixed
*R*[*F*^2^ > 2σ(*F*^2^)] = 0.035	H atoms treated by a mixture of independent and constrained refinement
*wR*(*F*^2^) = 0.083	*w* = 1/[σ^2^(*F*_o_^2^) + (0.0426*P*)^2^ + 1.0094*P*] where *P* = (*F*_o_^2^ + 2*F*_c_^2^)/3
*S* = 1.02	(Δ/σ)_max_ = 0.001
7124 reflections	Δρ_max_ = 0.65 e Å^−^^3^
394 parameters	Δρ_min_ = −0.36 e Å^−^^3^
2 restraints	

### Dichloridobis(N,N'-dicyclohexyl-3-cyclopropylprop-2-ynamidine)cobalt(II) (6) Special details

**Table:**

Geometry. All esds (except the esd in the dihedral angle between two l.s. planes) are estimated using the full covariance matrix. The cell esds are taken into account individually in the estimation of esds in distances, angles and torsion angles; correlations between esds in cell parameters are only used when they are defined by crystal symmetry. An approximate (isotropic) treatment of cell esds is used for estimating esds involving l.s. planes.

### Dichloridobis(N,N'-dicyclohexyl-3-cyclopropylprop-2-ynamidine)cobalt(II) (6) Fractional atomic coordinates and isotropic or equivalent isotropic displacement parameters (Å^2^)

**Table:**

	*x*	*y*	*z*	*U*_iso_*/*U*_eq_	
C1	0.68127 (13)	0.26772 (14)	0.01098 (8)	0.0221 (4)	
C2	0.68620 (13)	0.33626 (15)	−0.04462 (8)	0.0246 (4)	
C3	0.69022 (13)	0.39139 (14)	−0.09095 (8)	0.0238 (4)	
C4	0.69597 (14)	0.45734 (15)	−0.14694 (8)	0.0255 (4)	
H2	0.639622	0.453885	−0.177878	0.031*	
C5	0.74656 (15)	0.56342 (15)	−0.14185 (9)	0.0314 (4)	
H3	0.720164	0.623037	−0.168012	0.038*	
H4	0.774340	0.584306	−0.099389	0.038*	
C6	0.79318 (16)	0.47400 (17)	−0.17614 (10)	0.0357 (5)	
H5	0.849734	0.439518	−0.154864	0.043*	
H6	0.795549	0.478255	−0.223499	0.043*	
C7	0.84593 (13)	0.30851 (14)	0.03345 (8)	0.0216 (4)	
H7	0.829147	0.375167	0.009358	0.026*	
C8	0.90582 (13)	0.33917 (15)	0.09268 (8)	0.0242 (4)	
H8	0.923234	0.274419	0.117521	0.029*	
H9	0.867750	0.386219	0.120380	0.029*	
C9	0.99708 (14)	0.39662 (17)	0.07312 (9)	0.0311 (4)	
H11	0.979370	0.464254	0.051400	0.037*	
H10	1.036606	0.414083	0.112028	0.037*	
C10	1.05599 (15)	0.3288 (2)	0.02837 (10)	0.0386 (5)	
H13	1.111608	0.370733	0.013820	0.046*	
H12	1.081342	0.266023	0.052129	0.046*	
C11	0.99668 (15)	0.29143 (18)	−0.02980 (9)	0.0344 (5)	
H15	1.035268	0.240872	−0.054871	0.041*	
H14	0.980910	0.353300	−0.057618	0.041*	
C12	0.90331 (14)	0.23711 (15)	−0.01041 (8)	0.0275 (4)	
H17	0.863810	0.220094	−0.049399	0.033*	
H16	0.918831	0.169466	0.012003	0.033*	
C13	0.50831 (13)	0.24230 (15)	−0.01777 (8)	0.0251 (4)	
H18	0.525235	0.259981	−0.062842	0.030*	
C14	0.44241 (14)	0.14592 (16)	−0.01981 (9)	0.0293 (4)	
H19	0.427183	0.124519	0.024470	0.035*	
H20	0.475377	0.085598	−0.040437	0.035*	
C15	0.34929 (14)	0.17201 (16)	−0.05751 (9)	0.0303 (4)	
H22	0.364137	0.184913	−0.103020	0.036*	
H21	0.305130	0.110251	−0.055881	0.036*	
C16	0.29970 (14)	0.26938 (18)	−0.03075 (9)	0.0345 (5)	
H24	0.242165	0.286411	−0.058018	0.041*	
H23	0.277878	0.253451	0.013014	0.041*	
C17	0.36628 (16)	0.36497 (17)	−0.02816 (10)	0.0369 (5)	
H25	0.333240	0.425682	−0.007993	0.044*	
H26	0.382614	0.386012	−0.072302	0.044*	
C18	0.45860 (15)	0.33863 (16)	0.01048 (10)	0.0328 (4)	
H27	0.502652	0.400597	0.010098	0.039*	
H28	0.442784	0.323783	0.055614	0.039*	
C19	0.84880 (13)	0.27434 (14)	0.25053 (8)	0.0213 (4)	
C20	0.85597 (13)	0.34902 (14)	0.30319 (8)	0.0236 (4)	
C21	0.85812 (14)	0.41499 (15)	0.34448 (8)	0.0259 (4)	
C22	0.85834 (17)	0.49612 (17)	0.39270 (9)	0.0364 (5)	
H30	0.922987	0.522900	0.407681	0.044*	
C23	0.7774 (2)	0.57704 (18)	0.39069 (11)	0.0444 (6)	
H31	0.793351	0.651330	0.402858	0.053*	
H32	0.727111	0.569752	0.356306	0.053*	
C24	0.7808 (2)	0.4955 (2)	0.44195 (11)	0.0510 (7)	
H34	0.732534	0.437550	0.439489	0.061*	
H33	0.798758	0.519109	0.486029	0.061*	
C25	0.68956 (13)	0.33810 (15)	0.22933 (8)	0.0235 (4)	
H35	0.715959	0.403773	0.250527	0.028*	
C26	0.63157 (14)	0.37183 (15)	0.16962 (8)	0.0255 (4)	
H37	0.607557	0.307863	0.146445	0.031*	
H36	0.673432	0.412041	0.140594	0.031*	
C27	0.54659 (15)	0.44134 (17)	0.18814 (9)	0.0336 (5)	
H38	0.570996	0.508103	0.207974	0.040*	
H39	0.508123	0.460339	0.149046	0.040*	
C28	0.48268 (16)	0.3839 (2)	0.23505 (10)	0.0408 (5)	
H40	0.429548	0.431574	0.247395	0.049*	
H41	0.454034	0.320153	0.214098	0.049*	
C29	0.54070 (16)	0.3506 (2)	0.29479 (10)	0.0420 (6)	
H43	0.498800	0.310458	0.323824	0.050*	
H42	0.564340	0.414812	0.317882	0.050*	
C30	0.62621 (14)	0.28122 (17)	0.27699 (9)	0.0310 (4)	
H44	0.664916	0.263746	0.316245	0.037*	
H45	0.602324	0.213672	0.257887	0.037*	
C31	1.01589 (13)	0.21371 (14)	0.28045 (8)	0.0223 (4)	
H46	1.000389	0.228954	0.326094	0.027*	
C32	1.08503 (14)	0.29842 (16)	0.25785 (9)	0.0296 (4)	
H47	1.055450	0.369628	0.262519	0.036*	
H48	1.097814	0.287215	0.211865	0.036*	
C33	1.17997 (15)	0.29393 (16)	0.29703 (10)	0.0319 (4)	
H50	1.224839	0.348128	0.280692	0.038*	
H49	1.167746	0.310826	0.342464	0.038*	
C34	1.22609 (14)	0.18423 (17)	0.29290 (10)	0.0323 (4)	
H52	1.244067	0.170104	0.248086	0.039*	
H51	1.285534	0.182371	0.320300	0.039*	
C35	1.15711 (15)	0.09852 (16)	0.31466 (10)	0.0311 (4)	
H53	1.145601	0.107716	0.360982	0.037*	
H54	1.186802	0.027682	0.308657	0.037*	
C36	1.06089 (14)	0.10306 (15)	0.27715 (9)	0.0289 (4)	
H56	1.071126	0.084213	0.231730	0.035*	
H55	1.016155	0.050150	0.295016	0.035*	
N1	0.75490 (11)	0.25596 (12)	0.05117 (7)	0.0213 (3)	
N2	0.59761 (11)	0.21671 (13)	0.01815 (7)	0.0262 (3)	
H1	0.5925 (16)	0.1765 (16)	0.0506 (9)	0.031*	
N3	0.77203 (11)	0.26909 (11)	0.21253 (7)	0.0211 (3)	
N4	0.92585 (11)	0.21171 (13)	0.24215 (7)	0.0232 (3)	
H29	0.9211 (16)	0.1646 (15)	0.2125 (9)	0.028*	
Co	0.75507 (2)	0.17118 (2)	0.13474 (2)	0.02071 (7)	
Cl1	0.87506 (4)	0.04756 (3)	0.12921 (2)	0.02899 (11)	
Cl2	0.61692 (4)	0.07705 (4)	0.14621 (2)	0.03201 (11)	

### Dichloridobis(N,N'-dicyclohexyl-3-cyclopropylprop-2-ynamidine)cobalt(II) (6) Atomic displacement parameters (Å^2^)

**Table:**

	*U*^11^	*U*^22^	*U*^33^	*U*^12^	*U*^13^	*U*^23^
C1	0.0268 (9)	0.0219 (8)	0.0176 (8)	−0.0004 (7)	0.0012 (7)	−0.0010 (6)
C2	0.0229 (9)	0.0268 (9)	0.0240 (9)	−0.0021 (8)	−0.0024 (7)	−0.0011 (7)
C3	0.0233 (9)	0.0248 (9)	0.0234 (9)	−0.0012 (7)	−0.0011 (7)	−0.0010 (7)
C4	0.0279 (10)	0.0252 (9)	0.0232 (8)	−0.0019 (8)	−0.0026 (7)	0.0047 (7)
C5	0.0367 (11)	0.0265 (10)	0.0310 (10)	−0.0053 (9)	0.0014 (8)	0.0010 (8)
C6	0.0396 (12)	0.0349 (11)	0.0330 (10)	0.0008 (10)	0.0105 (9)	0.0058 (9)
C7	0.0220 (9)	0.0234 (9)	0.0196 (8)	−0.0034 (7)	0.0007 (7)	0.0013 (7)
C8	0.0263 (9)	0.0264 (9)	0.0199 (8)	−0.0044 (8)	0.0012 (7)	−0.0021 (7)
C9	0.0273 (10)	0.0412 (11)	0.0248 (9)	−0.0108 (9)	−0.0008 (7)	−0.0020 (8)
C10	0.0245 (10)	0.0573 (14)	0.0342 (11)	−0.0047 (10)	0.0040 (8)	−0.0021 (10)
C11	0.0308 (11)	0.0451 (12)	0.0277 (9)	−0.0036 (9)	0.0081 (8)	−0.0068 (9)
C12	0.0285 (10)	0.0314 (10)	0.0227 (9)	−0.0033 (8)	0.0038 (7)	−0.0042 (7)
C13	0.0232 (9)	0.0290 (9)	0.0227 (8)	−0.0016 (8)	−0.0029 (7)	0.0026 (7)
C14	0.0273 (10)	0.0281 (10)	0.0323 (10)	−0.0023 (8)	−0.0014 (8)	0.0014 (8)
C15	0.0238 (9)	0.0354 (10)	0.0316 (10)	−0.0052 (8)	−0.0024 (8)	0.0019 (8)
C16	0.0248 (10)	0.0504 (13)	0.0282 (10)	0.0069 (9)	0.0013 (8)	0.0012 (9)
C17	0.0363 (12)	0.0352 (11)	0.0389 (11)	0.0105 (10)	−0.0014 (9)	−0.0026 (9)
C18	0.0343 (11)	0.0303 (10)	0.0335 (10)	0.0003 (9)	−0.0027 (8)	−0.0062 (8)
C19	0.0250 (9)	0.0219 (8)	0.0171 (8)	0.0009 (7)	0.0027 (7)	0.0018 (6)
C20	0.0242 (9)	0.0256 (9)	0.0211 (8)	0.0020 (8)	0.0006 (7)	0.0014 (7)
C21	0.0282 (10)	0.0280 (9)	0.0214 (8)	0.0023 (8)	−0.0002 (7)	0.0014 (7)
C22	0.0416 (12)	0.0371 (11)	0.0304 (10)	0.0013 (10)	−0.0008 (9)	−0.0133 (9)
C23	0.0635 (16)	0.0295 (11)	0.0404 (12)	0.0109 (11)	0.0054 (11)	−0.0069 (9)
C24	0.0733 (18)	0.0509 (14)	0.0297 (11)	0.0245 (13)	0.0150 (11)	−0.0016 (10)
C25	0.0246 (9)	0.0261 (9)	0.0200 (8)	0.0056 (8)	0.0010 (7)	0.0001 (7)
C26	0.0274 (10)	0.0292 (9)	0.0201 (8)	0.0069 (8)	0.0017 (7)	0.0026 (7)
C27	0.0367 (11)	0.0393 (11)	0.0247 (9)	0.0175 (9)	−0.0003 (8)	0.0022 (8)
C28	0.0305 (11)	0.0610 (15)	0.0313 (10)	0.0196 (11)	0.0069 (8)	0.0055 (10)
C29	0.0371 (12)	0.0635 (15)	0.0260 (10)	0.0199 (11)	0.0098 (9)	0.0089 (10)
C30	0.0280 (10)	0.0415 (11)	0.0238 (9)	0.0102 (9)	0.0046 (8)	0.0091 (8)
C31	0.0243 (9)	0.0239 (8)	0.0185 (8)	0.0026 (7)	−0.0019 (7)	0.0002 (7)
C32	0.0300 (10)	0.0267 (9)	0.0318 (10)	−0.0011 (8)	−0.0043 (8)	0.0069 (8)
C33	0.0287 (10)	0.0299 (10)	0.0366 (10)	−0.0046 (9)	−0.0070 (8)	0.0046 (8)
C34	0.0242 (10)	0.0399 (11)	0.0324 (10)	0.0027 (9)	−0.0030 (8)	−0.0040 (8)
C35	0.0306 (10)	0.0259 (9)	0.0365 (10)	0.0079 (8)	−0.0057 (8)	−0.0002 (8)
C36	0.0280 (10)	0.0233 (9)	0.0353 (10)	0.0018 (8)	−0.0021 (8)	−0.0022 (8)
N1	0.0217 (8)	0.0223 (7)	0.0198 (7)	−0.0024 (6)	0.0009 (6)	0.0000 (6)
N2	0.0230 (8)	0.0312 (8)	0.0242 (7)	−0.0041 (7)	−0.0040 (6)	0.0077 (6)
N3	0.0236 (8)	0.0213 (7)	0.0183 (7)	0.0027 (6)	0.0003 (6)	0.0023 (6)
N4	0.0241 (8)	0.0253 (8)	0.0200 (7)	0.0034 (7)	−0.0027 (6)	−0.0052 (6)
Co	0.02311 (13)	0.02012 (12)	0.01881 (12)	−0.00067 (10)	−0.00071 (9)	0.00079 (9)
Cl1	0.0338 (3)	0.0225 (2)	0.0304 (2)	0.00481 (19)	−0.00324 (18)	−0.00277 (17)
Cl2	0.0329 (3)	0.0295 (2)	0.0336 (2)	−0.0093 (2)	0.00000 (19)	0.00584 (18)

### Dichloridobis(N,N'-dicyclohexyl-3-cyclopropylprop-2-ynamidine)cobalt(II) (6) Geometric parameters (Å, º)

**Table:**

C1—N1	1.311 (2)	C20—C21	1.194 (3)
C1—N2	1.339 (2)	C21—C22	1.431 (3)
C1—C2	1.447 (2)	C22—C24	1.510 (3)
C2—C3	1.191 (3)	C22—C23	1.515 (3)
C3—C4	1.435 (2)	C22—H30	1.0000
C4—C5	1.508 (3)	C23—C24	1.479 (3)
C4—C6	1.512 (3)	C23—H31	0.9900
C4—H2	1.0000	C23—H32	0.9900
C5—C6	1.490 (3)	C24—H34	0.9900
C5—H3	0.9900	C24—H33	0.9900
C5—H4	0.9900	C25—N3	1.487 (2)
C6—H5	0.9900	C25—C26	1.522 (2)
C6—H6	0.9900	C25—C30	1.524 (2)
C7—N1	1.483 (2)	C25—H35	1.0000
C7—C8	1.517 (2)	C26—C27	1.527 (3)
C7—C12	1.523 (2)	C26—H37	0.9900
C7—H7	1.0000	C26—H36	0.9900
C8—C9	1.525 (3)	C27—C28	1.522 (3)
C8—H8	0.9900	C27—H38	0.9900
C8—H9	0.9900	C27—H39	0.9900
C9—C10	1.520 (3)	C28—C29	1.521 (3)
C9—H11	0.9900	C28—H40	0.9900
C9—H10	0.9900	C28—H41	0.9900
C10—C11	1.519 (3)	C29—C30	1.528 (3)
C10—H13	0.9900	C29—H43	0.9900
C10—H12	0.9900	C29—H42	0.9900
C11—C12	1.530 (3)	C30—H44	0.9900
C11—H15	0.9900	C30—H45	0.9900
C11—H14	0.9900	C31—N4	1.463 (2)
C12—H17	0.9900	C31—C32	1.517 (3)
C12—H16	0.9900	C31—C36	1.526 (3)
C13—N2	1.465 (2)	C31—H46	1.0000
C13—C14	1.517 (3)	C32—C33	1.531 (3)
C13—C18	1.520 (3)	C32—H47	0.9900
C13—H18	1.0000	C32—H48	0.9900
C14—C15	1.529 (3)	C33—C34	1.523 (3)
C14—H19	0.9900	C33—H50	0.9900
C14—H20	0.9900	C33—H49	0.9900
C15—C16	1.518 (3)	C34—C35	1.519 (3)
C15—H22	0.9900	C34—H52	0.9900
C15—H21	0.9900	C34—H51	0.9900
C16—C17	1.515 (3)	C35—C36	1.529 (3)
C16—H24	0.9900	C35—H53	0.9900
C16—H23	0.9900	C35—H54	0.9900
C17—C18	1.530 (3)	C36—H56	0.9900
C17—H25	0.9900	C36—H55	0.9900
C17—H26	0.9900	N1—Co	2.0412 (14)
C18—H27	0.9900	N2—H1	0.848 (15)
C18—H28	0.9900	N3—Co	2.0426 (14)
C19—N3	1.311 (2)	N4—H29	0.856 (15)
C19—N4	1.344 (2)	Co—Cl2	2.2725 (5)
C19—C20	1.445 (2)	Co—Cl1	2.2830 (5)
			
N1—C1—N2	122.55 (16)	C24—C22—H30	116.2
N1—C1—C2	121.60 (16)	C23—C22—H30	116.2
N2—C1—C2	115.85 (16)	C24—C23—C22	60.54 (15)
C3—C2—C1	179.04 (19)	C24—C23—H31	117.7
C2—C3—C4	179.4 (2)	C22—C23—H31	117.7
C3—C4—C5	119.33 (16)	C24—C23—H32	117.7
C3—C4—C6	118.63 (17)	C22—C23—H32	117.7
C5—C4—C6	59.10 (13)	H31—C23—H32	114.8
C3—C4—H2	116.0	C23—C24—C22	60.89 (16)
C5—C4—H2	116.0	C23—C24—H34	117.7
C6—C4—H2	116.0	C22—C24—H34	117.7
C6—C5—C4	60.58 (13)	C23—C24—H33	117.7
C6—C5—H3	117.7	C22—C24—H33	117.7
C4—C5—H3	117.7	H34—C24—H33	114.8
C6—C5—H4	117.7	N3—C25—C26	111.25 (14)
C4—C5—H4	117.7	N3—C25—C30	110.13 (15)
H3—C5—H4	114.8	C26—C25—C30	111.19 (16)
C5—C6—C4	60.33 (13)	N3—C25—H35	108.0
C5—C6—H5	117.7	C26—C25—H35	108.0
C4—C6—H5	117.7	C30—C25—H35	108.0
C5—C6—H6	117.7	C25—C26—C27	110.28 (14)
C4—C6—H6	117.7	C25—C26—H37	109.6
H5—C6—H6	114.9	C27—C26—H37	109.6
N1—C7—C8	111.17 (13)	C25—C26—H36	109.6
N1—C7—C12	110.67 (14)	C27—C26—H36	109.6
C8—C7—C12	110.71 (15)	H37—C26—H36	108.1
N1—C7—H7	108.1	C28—C27—C26	111.21 (17)
C8—C7—H7	108.1	C28—C27—H38	109.4
C12—C7—H7	108.1	C26—C27—H38	109.4
C7—C8—C9	110.03 (14)	C28—C27—H39	109.4
C7—C8—H8	109.7	C26—C27—H39	109.4
C9—C8—H8	109.7	H38—C27—H39	108.0
C7—C8—H9	109.7	C29—C28—C27	110.56 (19)
C9—C8—H9	109.7	C29—C28—H40	109.5
H8—C8—H9	108.2	C27—C28—H40	109.5
C10—C9—C8	111.47 (17)	C29—C28—H41	109.5
C10—C9—H11	109.3	C27—C28—H41	109.5
C8—C9—H11	109.3	H40—C28—H41	108.1
C10—C9—H10	109.3	C28—C29—C30	110.79 (16)
C8—C9—H10	109.3	C28—C29—H43	109.5
H11—C9—H10	108.0	C30—C29—H43	109.5
C11—C10—C9	111.94 (18)	C28—C29—H42	109.5
C11—C10—H13	109.2	C30—C29—H42	109.5
C9—C10—H13	109.2	H43—C29—H42	108.1
C11—C10—H12	109.2	C25—C30—C29	111.05 (17)
C9—C10—H12	109.2	C25—C30—H44	109.4
H13—C10—H12	107.9	C29—C30—H44	109.4
C10—C11—C12	111.75 (16)	C25—C30—H45	109.4
C10—C11—H15	109.3	C29—C30—H45	109.4
C12—C11—H15	109.3	H44—C30—H45	108.0
C10—C11—H14	109.3	N4—C31—C32	112.41 (14)
C12—C11—H14	109.3	N4—C31—C36	107.71 (15)
H15—C11—H14	107.9	C32—C31—C36	111.12 (16)
C7—C12—C11	111.04 (16)	N4—C31—H46	108.5
C7—C12—H17	109.4	C32—C31—H46	108.5
C11—C12—H17	109.4	C36—C31—H46	108.5
C7—C12—H16	109.4	C31—C32—C33	110.59 (15)
C11—C12—H16	109.4	C31—C32—H47	109.5
H17—C12—H16	108.0	C33—C32—H47	109.5
N2—C13—C14	109.88 (15)	C31—C32—H48	109.5
N2—C13—C18	111.35 (15)	C33—C32—H48	109.5
C14—C13—C18	111.41 (16)	H47—C32—H48	108.1
N2—C13—H18	108.0	C34—C33—C32	111.12 (16)
C14—C13—H18	108.0	C34—C33—H50	109.4
C18—C13—H18	108.0	C32—C33—H50	109.4
C13—C14—C15	110.11 (16)	C34—C33—H49	109.4
C13—C14—H19	109.6	C32—C33—H49	109.4
C15—C14—H19	109.6	H50—C33—H49	108.0
C13—C14—H20	109.6	C35—C34—C33	110.73 (16)
C15—C14—H20	109.6	C35—C34—H52	109.5
H19—C14—H20	108.2	C33—C34—H52	109.5
C16—C15—C14	111.73 (16)	C35—C34—H51	109.5
C16—C15—H22	109.3	C33—C34—H51	109.5
C14—C15—H22	109.3	H52—C34—H51	108.1
C16—C15—H21	109.3	C34—C35—C36	111.74 (16)
C14—C15—H21	109.3	C34—C35—H53	109.3
H22—C15—H21	107.9	C36—C35—H53	109.3
C17—C16—C15	111.59 (16)	C34—C35—H54	109.3
C17—C16—H24	109.3	C36—C35—H54	109.3
C15—C16—H24	109.3	H53—C35—H54	107.9
C17—C16—H23	109.3	C31—C36—C35	111.34 (16)
C15—C16—H23	109.3	C31—C36—H56	109.4
H24—C16—H23	108.0	C35—C36—H56	109.4
C16—C17—C18	110.40 (17)	C31—C36—H55	109.4
C16—C17—H25	109.6	C35—C36—H55	109.4
C18—C17—H25	109.6	H56—C36—H55	108.0
C16—C17—H26	109.6	C1—N1—C7	116.47 (14)
C18—C17—H26	109.6	C1—N1—Co	125.79 (12)
H25—C17—H26	108.1	C7—N1—Co	117.74 (11)
C13—C18—C17	110.58 (16)	C1—N2—C13	124.21 (15)
C13—C18—H27	109.5	C1—N2—H1	117.9 (16)
C17—C18—H27	109.5	C13—N2—H1	116.6 (16)
C13—C18—H28	109.5	C19—N3—C25	116.56 (14)
C17—C18—H28	109.5	C19—N3—Co	125.60 (12)
H27—C18—H28	108.1	C25—N3—Co	117.81 (11)
N3—C19—N4	121.93 (16)	C19—N4—C31	126.16 (15)
N3—C19—C20	121.86 (16)	C19—N4—H29	117.0 (15)
N4—C19—C20	116.20 (16)	C31—N4—H29	116.8 (15)
C21—C20—C19	175.8 (2)	N1—Co—N3	111.12 (6)
C20—C21—C22	178.0 (2)	N1—Co—Cl2	112.43 (4)
C21—C22—C24	119.2 (2)	N3—Co—Cl2	107.90 (4)
C21—C22—C23	118.18 (19)	N1—Co—Cl1	107.08 (4)
C24—C22—C23	58.57 (15)	N3—Co—Cl1	112.48 (4)
C21—C22—H30	116.2	Cl2—Co—Cl1	105.76 (2)
			
C3—C4—C5—C6	107.7 (2)	C31—C32—C33—C34	57.3 (2)
C3—C4—C6—C5	−108.88 (19)	C32—C33—C34—C35	−56.4 (2)
N1—C7—C8—C9	−177.91 (15)	C33—C34—C35—C36	55.0 (2)
C12—C7—C8—C9	58.6 (2)	N4—C31—C36—C35	178.57 (15)
C7—C8—C9—C10	−57.1 (2)	C32—C31—C36—C35	55.0 (2)
C8—C9—C10—C11	54.2 (2)	C34—C35—C36—C31	−54.4 (2)
C9—C10—C11—C12	−52.3 (3)	N2—C1—N1—C7	173.79 (16)
N1—C7—C12—C11	179.12 (15)	C2—C1—N1—C7	−5.4 (2)
C8—C7—C12—C11	−57.1 (2)	N2—C1—N1—Co	−5.6 (2)
C10—C11—C12—C7	53.7 (2)	C2—C1—N1—Co	175.14 (12)
N2—C13—C14—C15	−179.74 (15)	C8—C7—N1—C1	150.77 (16)
C18—C13—C14—C15	56.4 (2)	C12—C7—N1—C1	−85.76 (19)
C13—C14—C15—C16	−55.0 (2)	C8—C7—N1—Co	−29.76 (18)
C14—C15—C16—C17	55.4 (2)	C12—C7—N1—Co	93.71 (15)
C15—C16—C17—C18	−55.7 (2)	N1—C1—N2—C13	167.74 (17)
N2—C13—C18—C17	179.27 (16)	C2—C1—N2—C13	−13.0 (3)
C14—C13—C18—C17	−57.7 (2)	C14—C13—N2—C1	156.28 (17)
C16—C17—C18—C13	56.6 (2)	C18—C13—N2—C1	−79.8 (2)
C21—C22—C23—C24	108.7 (2)	N4—C19—N3—C25	176.46 (15)
C21—C22—C24—C23	−107.0 (2)	C20—C19—N3—C25	−4.6 (2)
N3—C25—C26—C27	179.25 (16)	N4—C19—N3—Co	−1.4 (2)
C30—C25—C26—C27	56.1 (2)	C20—C19—N3—Co	177.62 (12)
C25—C26—C27—C28	−56.9 (2)	C26—C25—N3—C19	149.59 (16)
C26—C27—C28—C29	57.3 (2)	C30—C25—N3—C19	−86.66 (19)
C27—C28—C29—C30	−56.5 (3)	C26—C25—N3—Co	−32.41 (18)
N3—C25—C30—C29	−179.81 (16)	C30—C25—N3—Co	91.34 (15)
C26—C25—C30—C29	−56.0 (2)	N3—C19—N4—C31	177.89 (16)
C28—C29—C30—C25	56.0 (3)	C20—C19—N4—C31	−1.1 (3)
N4—C31—C32—C33	−177.17 (15)	C32—C31—N4—C19	−83.8 (2)
C36—C31—C32—C33	−56.4 (2)	C36—C31—N4—C19	153.48 (17)

### Dichloridobis(N,N'-dicyclohexyl-3-cyclopropylprop-2-ynamidine)cobalt(II) (6) Hydrogen-bond geometry (Å, º)

**Table:**

*D*—H···*A*	*D*—H	H···*A*	*D*···*A*	*D*—H···*A*
N2—H1···Cl2	0.85 (2)	2.37 (2)	3.1979 (16)	166 (2)
N4—H29···Cl1	0.86 (2)	2.35 (2)	3.1917 (15)	168 (2)
C22—H30···Cl1^i^	1.00	2.95	3.800 (2)	144
C33—H49···Cl1^i^	0.99	3.09	3.628 (2)	115
C28—H40···Cl2^ii^	0.99	2.96	3.758 (2)	139

Symmetry codes: (i) −*x*+2, *y*+1/2, −*z*+1/2; (ii) −*x*+1, *y*+1/2, −*z*+1/2.
